# Glucocorticoid-mediated induction of caveolin-1 disrupts cytoskeletal organization, inhibits cell migration and re-epithelialization of non-healing wounds

**DOI:** 10.1038/s42003-021-02298-5

**Published:** 2021-06-18

**Authors:** Ivan Jozic, Beatriz Abdo Abujamra, Michael H. Elliott, Tongyu C. Wikramanayake, Jelena Marjanovic, Rivka C. Stone, Cheyanne R. Head, Irena Pastar, Robert S. Kirsner, Fotios M. Andreopoulos, Juan P. Musi, Marjana Tomic-Canic

**Affiliations:** 1 Wound Healing and Regenerative Medicine Research Program, Department of Dermatology and Cutaneous Surgery, University of Miami Miller School of Medicine, Miami, FL USA; 2 Departments of Ophthalmology, Physiology, and Oklahoma Center for Neuroscience, University of Oklahoma Health Sciences Center, Oklahoma City, OK USA; 3 Department of Biomedical Engineering, University of Miami, Coral Gables, FL USA; 4 Department of Surgery, University of Miami Miller School of Medicine, Miami, FL USA; 5 John P. Hussman Institute for Human Genomics, University of Miami Miller School of Medicine, Miami, FL USA

**Keywords:** Actin, Translational research, Skin diseases

## Abstract

Although impaired keratinocyte migration is a recognized hallmark of chronic wounds, the molecular mechanisms underpinning impaired cell movement are poorly understood. Here, we demonstrate that both diabetic foot ulcers (DFUs) and venous leg ulcers (VLUs) exhibit global deregulation of cytoskeletal organization in genomic comparison to normal skin and acute wounds. Interestingly, we found that DFUs and VLUs exhibited downregulation of ArhGAP35, which serves both as an inactivator of RhoA and as a glucocorticoid repressor. Since chronic wounds exhibit elevated levels of cortisol and caveolin-1 (Cav1), we posited that observed elevation of Cav1 expression may contribute to impaired actin-cytoskeletal signaling, manifesting in aberrant keratinocyte migration. We showed that Cav1 indeed antagonizes ArhGAP35, resulting in increased activation of RhoA and diminished activation of Cdc42, which can be rescued by Cav1 disruption. Furthermore, we demonstrate that both inducible keratinocyte specific Cav1 knockout mice, and MβCD treated diabetic mice, exhibit accelerated wound closure. Taken together, our findings provide a previously unreported mechanism by which Cav1-mediated cytoskeletal organization prevents wound closure in patients with chronic wounds.

## Abstract (web summary)

Jozic et al observe deregulated cytoskeleton components and elevated levels of cortisol and caveolin-1 in chronic wounds. They also show that inducible-keratinocyte specific Cav1 knockout or drug-induced cholesterol disruption in diabetic mice leads to accelerated wound closure suggesting a therapeutic approach for diabetic foot ulcers.

## Introduction

Over 8 million Americans suffer from chronic wounds annually and this number is likely to rise with the aging population and the increasing incidence of obesity and diabetes^1^. Lack of understanding regarding the molecular mechanisms underpinning impaired healing in chronic wounds leads to increased mortality and serious co-morbidities including frequent lower leg amputations^2–6^. A very important component of the repair response mechanism is tight control of cell shape, recruitment of the necessary repair machinery and directional cellular migration, all of which are closely orchestrated by a network of cytoskeletal proteins^7–10^. In addition, impaired re-epithelialization is a well-recognized contributing factor to chronic wounds^11^, which occurs, in part, due to increased production of cortisol^12^. However, the exact role of the cytoskeletal reorganization in either acute or chronic wounds remains to be elucidated.

Rho family GTPases regulate the cytoskeletal changes necessary for formation of filopodia, lamellipodia, stress fibers and the resulting directional cell migration that underpins successful wound closure^13,14^. Thus, it is not surprising that they have been garnering attention in areas of wound repair and regeneration^15,16^. As with other small GTPases, Rho family of proteins are activated by guanine nucleotide exchange factors (GEFs), which facilitate exchange of GDP for GTP, and inactivated by GTPase accelerating proteins (GAPs), which stimulate their intrinsic GTPase activity. Although 22 members of the mammalian Rho family have been identified, three have garnered majority of the attention and research: Cdc42, Rac1 and RhoA.

One of the potential mechanisms by which Rho proteins can be regulated is by localizing to caveolae and interacting with caveolins^14^. Caveolae are cholesterol- and sphingolipid-rich microdomains in the plasma membrane which allow compartmentalization and clustering of signaling molecules^17,18^, with caveolin serving as the integral membrane protein essential for caveolar formation^18,19^. Caveolin binds various structural and signaling molecules that contain a caveolin binding domain^19^ and in doing so, it has been implicated in a variety of cellular processes ranging from vesicular transport, internalization of pathogens, and integration of signaling pathways and regulation of cell proliferation (reviewed in^17,20^). Through its scaffolding domain, caveolin-1 (Cav1) acts to sequester and compartmentalize these signal transduction molecules, thereby affording orchestration of transmembrane signaling events and allowing crosstalk between various downstream effectors^21,22^. We have shown previously that Cav1 localizes to basal keratinocytes and is spatiotemporally downregulated during acute wound healing, suggesting an inhibitory role during cutaneous wound re-epithelialization^12,15^. Furthermore, we have also shown that topical application of cholesterol depleting agents (methyl-β-cycloxextrin (MβCD) or mevastatin) accelerated wound closure of human skin ex vivo as well as murine and porcine skin in vivo^12,15^. Since MβCD disrupts caveolae by removing cholesterol from the membrane, these observations are in line with an observation that cholesterol synthesis decreases as a function of distance from the wound edge in human acute wounds, which would thus posit spatiotemporal Cav1 downregulation in the healing wound^23^. Interestingly, we have also observed aberrant upregulation of Cav1 expression in non-healing diabetic foot ulcers and venous leg ulcers, two different types of chronic wounds^12^.

We demonstrated that chronic wounds exhibit elevated levels of cortisol which in turn induces expression of Cav1^12^ and thus provides a direct link between a stress-related hormone, Cav1 and inhibition of epithelialization. We established that the membrane fraction of the glucocorticoid receptor (mbGR) and its complex with Cav1 contributes to GC-mediated inhibition of keratinocyte migration^24^. Consequently, we focused on the mechanism by which increased cortisol synthesis impacts keratinocyte migration in DFUs. We hypothesized that cortisol-mediated upregulation of Cav1 in chronic wounds may act to sequester various signaling molecules, including Rho GTPases, to inhibit directional keratinocyte migration and subsequent wound closure. We used in vitro, human ex vivo, and human and mouse in vivo approaches to test our hypothesis. Here, we show that both DFUs and VLUs exhibit global deregulation of actin-based cytoskeletal machinery. Furthermore, we show that increased production of GCs in DFUs leads to a downregulation of a glucocorticoid receptor repressor ArhGAP35 (aka GRLF1). Subsequently, we show that GCs stimulate Cav1 expression but inhibit ArhGAP35 expression, leading to increased stress fiber formation. This increase of stress fibers is a result of Cav1 associated selective activation of RhoA and a concomitant inactivation of Cdc42. Conversely, disruption of Cav1 (by MβCD) or knockout of Cav1 (by CRISPR/Cas9) reverses these GC-mediated events. Lastly, we validate these findings functionally in vivo by showing that selective downregulation of Cav1 in keratinocytes (using ^KRT14^Cav1^KO^ mouse model) accelerates cutaneous re-epithelialization in vivo and that Cav1 disruption reverses delayed wound epithelialization in the db/db mouse wound model. Together, these data provide mechanistic insights regarding Cav1-mediated inhibition of wound closure observed in patients and provide pre-clinical testing for therapeutic interventions targeting by disruption of cholesterol.

## Results

### Expression of cytoskeletal genes is significantly altered in chronic wounds

**Fig. 1:**
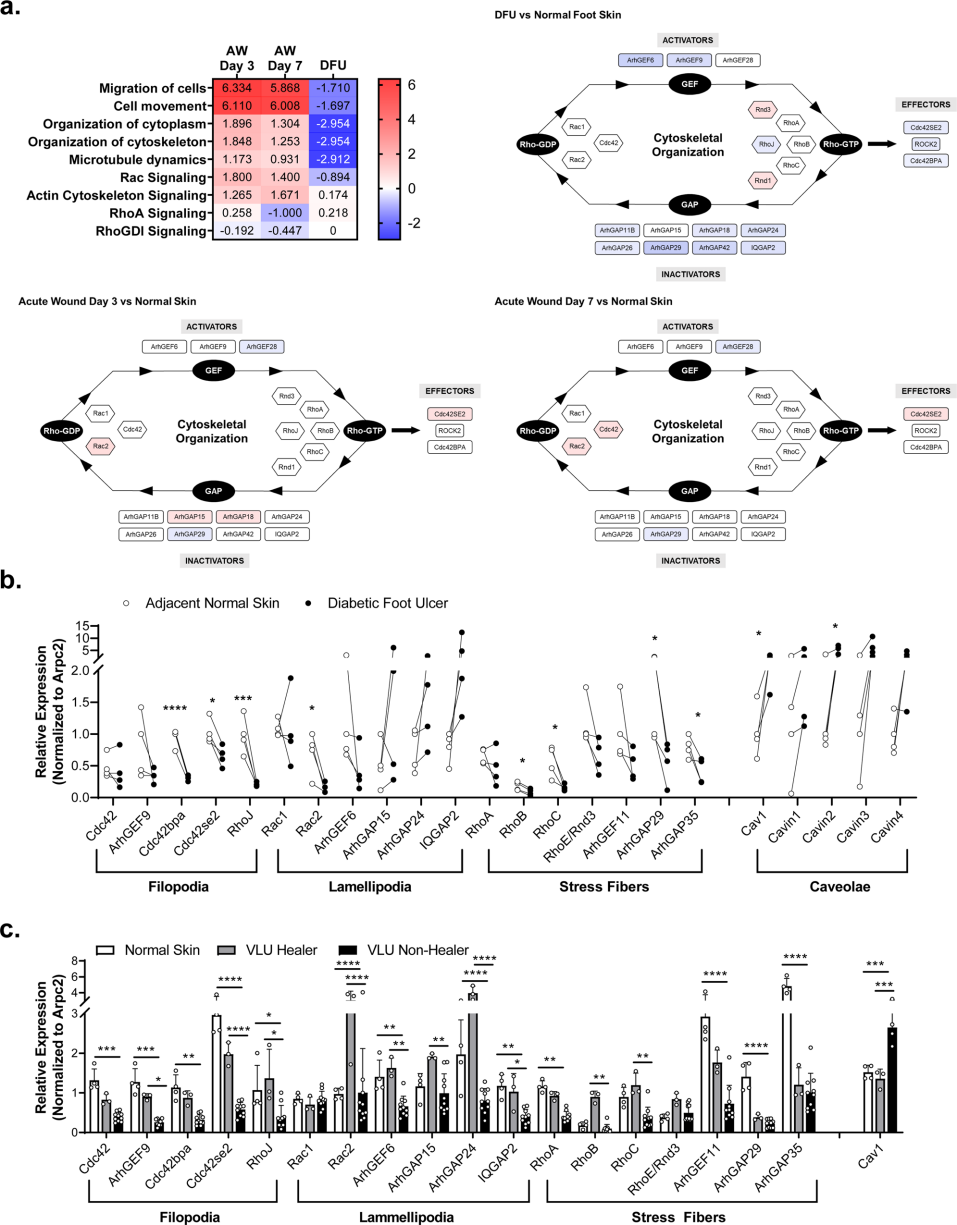
Global deregulation of cytoskeletal machinery in chronic wounds. **a** IPA analysis of global gene expression in DFUs in comparison to acute wounds predicts inhibition of cellular migration as expected and points to deregulation of cytoskeletal organization and the associated signaling through the Rho family of small GTPases (blue indicates downregulation, red indicates upregulation). **b** Levels of various actin-based cytoskeletal genes and structural components of caveolae were validated by qRT-PCR in DFU biopsies as well as adjacent normal skin from the same patient. *n* = 4 independent biological specimens, *p < 0.05, **p < 0.005 using 2-way ANOVA with Holm–Sidak correction for multiple comparisons. **c** Levels of same actin-based cytoskeletal genes were assessed by qRT-PCR in normal skin and compared to biopsies from VLU patients with a known healing outcome. Non-healing VLUs were defined as those whose ulcer size did not decrease by more than 40% after 8 weeks of standard of care (compression therapy)^12,27,28^. Error bars correspond to standard deviations from 17 biological specimens with statistical significance assessed using 2-way ANOVA with Tukey correction for multiple comparisons, *p < 0.05, **p < 0.005, ***p < 0.001, ****p < 0.0001.

To assess how are cytoskeletal genes affected in chronic wounds, we utilized Ingenuity Pathway Analysis (IPA) software to compare expression profiles of DFUs to human acute wounds at days 3 and 7 post wounding^25,26^. As expected, IPA predicted cellular movement and migration to be among the major processes deregulated in DFUs. We found both to be upregulated in acute wound and downregulated in DFUs (Fig. 1a). Furthermore, we observed stark differences in cytoskeletal organization and identified a subset of genes as the potential driving force responsible for deregulated cytoskeleton including members of Rho family of GTPases (Cdc42, Rac1, Rac2, RhoA, RhoB, RhoC, RhoJ, Rnd1, Rnd3/RhoE), and their respective activators (ArhGEF-6, -9, -11, -28), inactivators (ArhGAP-11B, -15, -18, -24, -26, -29, -35, -42 and IQGAP2) and downstream effectors (Cdc42SE2, Cdc42BPA, ROCK2) (Fig. 1a).

**Fig. 2:**
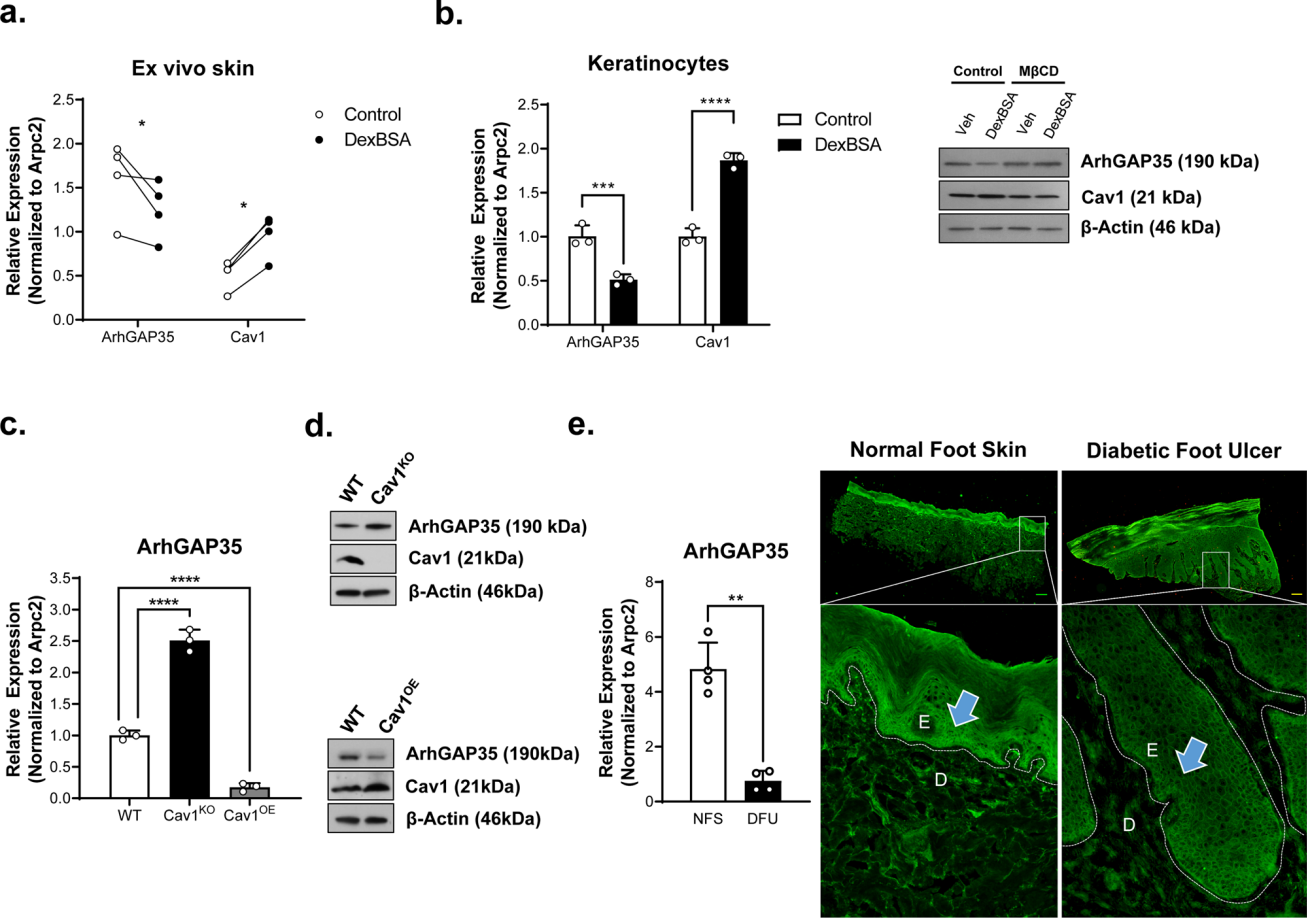
Glucocorticoids induce expression of Cav1 which antagonizes ArhGAP35. **a** Human ex vivo skin was treated ±DexBSA and levels of ArhGAP35 and Cav1 assessed by qRT-PCR. *n* = 4 independent biological specimens, *p < 0.05 using 2-way ANOVA with Holm–Sidak correction for multiple comparisons. **b** Human keratinocytes were treated ±DexBSA and levels of ArhGAP35 and Cav1 assessed by qRT-PCR and western blotting in presence or absence of MβCD. Error bars correspond to standard deviations from 3 independent experiments with statistical significance assessed using 2-way ANOVA with Holm–Sidak correction for multiple comparisons, ***p < 0.001, ****p < 0.0001. Relative protein levels were from western blots were semi-quantitated and included below each blot. ArhGAP35 was assessed by qRT-PCR (**c**) and western blotting (**d**) in human keratinocytes expressing endogenous levels of Cav1 (WT), Cav1-knockout cells (Cav1^KO^) and Cav1 overexpressing cells (Cav1^OE^). Error bars correspond to standard deviations from 3 independent experiments with statistical significance assessed using One-way ANOVA with Tukey correction for multiple comparisons, ****p < 0.0001. Relative protein levels were from western blots were semi-quantitated and included below each blot. **e** ArhGAP35 expression was assessed in normal foot skin (NFS) and diabetic foot ulcers (DFU) by qRT-PCR and validated by immunohistochemistry staining. Error bars correspond to standard deviations from 4 biological specimens with statistical significance assessed using paired *t*-test, **p < 0.005.

To further confirm results from IPA analyses, we analyzed expression of cytoskeletal genes in biopsies from DFUs and VLUs^12,27,28^. We found a statistically significant deregulation of a number of genes that correspond to formation of filopodia (Cdc42BPA, Cdc42SE2, RhoJ), lamellipodia (Rac2) and stress fibers (RhoC, ArhGAP29, ArhGAP35) in DFU biopsies when compared to location matched control skin sample from the same patient (Fig. 1b). As expected, we also observed an upregulation of Cav1 in DFU samples in comparison to the adjacent normal skin from the same patient (Fig. 1b). Next, we tested if this effect was specific to Cav1 or if it applies to other structural components of caveolae including the members of the Cavin family (Cavin1-4). Although we observed a trend towards upregulation of Cavin genes, we only observed Cavin2 to be elevated in a statistically significant manner (*p < 0.05, 2way ANOVA) (Fig. 2b).

Next, to test how levels of Cav1 and cytoskeletal gene expression are linked to clinical outcomes, we stratified VLU samples based on their surrogate clinical endpoints. Briefly, non-healing VLUs were defined as those whose ulcer size did not decrease by more than 40% after 8 weeks of standard of care (compression therapy)^12,27,28^. As expected, expression of Cav1 in non-healing VLU samples was significantly increased in comparison to control skin or healing VLU samples (Fig. 1c), as previously reported^12^. Non-healing VLUs showed deregulation of genes corresponding to functioning of filopodia (Cdc42, ArhGEF9, Cdc42BPA, Cdc42SE2, RhoJ), lamellipodia (Rac2, ArhGEF6, ArhGAP24, IQGAP2) and stress fibers (RhoA, ArhGEF11, ArhGAP29, ArhGAP35) (Fig. 1c) when compared to normal skin, whereas when comparing healing vs non-healing VLUs, we observed significant differences in expression of ArhGEF9, Cdc42SE2, RhoJ, Rac2, ArhGAP15, ArhGAP24, IQGAP2, RhoB and RhoC (Fig. 1c). Together, these data suggest that two different types of chronic wounds exhibit a similar deregulation of actin-based cytoskeletal machinery, which in turn contributes to cellular structure and affects their ability to migrate.

### Increased cortisol production promotes upregulation of Cav1 expression and downregulation of GR-repressor and RhoA-GAP, ArhGAP35

To further decipher the mechanism of GC-Cav1-mediated inhibition of epithelialization, we employed BSA-conjugated form of dexamethasone (DexBSA) to specifically target mbGR in topically treated human ex vivo wounds^12,15,24^. We used dexamethasone (Dex) as a representative of GC because it is a synthetic corticosteroid which is a poor substrate for HSD11β2 (the enzyme responsible for conversion of active cortisol into inactive cortisone), maintaining activity upon administration^25^ that recapitulate the chronic wound environment of excess cortisol presence. First, we quantified expression of ArhGAP35 (aka GRLF1 or p190RhoGAP) that functions as GR-repressor and is also a GTPase activating protein for RhoA^29,30^. We found that activation of mbGR inhibits expression of ArhGAP35 (Fig. 2a), whereas it upregulates Cav1 expression (Fig. 2a). We further substantiated these DexBSA-mediated effects specifically in keratinocytes (HaCaT cells) and confirmed downregulation of ArhGAP35 and upregulation of Cav1 expression on both mRNA and protein levels (Fig. 2b), similarly to human ex vivo experiments. Next, to test if Cav1 contributes to inhibition of ArhGAP35, we generated HaCaT Cav1 knockout cells (Cav1^KO^) using CRISPR/Cas9 and HaCaT overexpressing Cav1 cells (Cav1^OE^) using lentiviral infection, and quantified expression of ArhGAP35 at mRNA and protein levels. We found elevated levels of ArhGAP35 expression in Cav1^KO^ cells. Conversely, a downregulation of ArhGAP35 expression on both mRNA (Fig. 2c) and protein levels (Fig. 2d) was found in Cav1^OE^ cells, suggesting that Cav1 participates in regulation of a RhoA-GAP ArhGAP35 expression.

To further test the clinical relevance, we quantified expression of ArhGAP35 in the wound edge biopsies from patients with DFUs. As expected, ArhGAP35 was significantly downregulated at both mRNA and protein levels relative to location matched healthy foot skin (Fig. 2e). Together, these data suggest that elevated cortisol levels serve as Cav1 agonists, inducing its expression, which in turn contribute to downregulation of ArhGAP35 expression in human skin.

### Induction of cytoskeletal remodeling and formation of stress fibers by GCs is Cav1-dependent

To test how mbGR-Cav1 activation contributes to reorganization of actin cytoskeleton, we utilized DexBSA in the presence or absence of cholesterol depleting agent MβCD, and assayed actin dynamics by phalloidin staining in primary human keratinocytes. DexBSA induced stress fibers (Supplementary Fig. 1a) similar to calpeptin (CN01), a known inducer of stress fibers. Conversely, cholesterol depletion by MβCD led to actin reorganization, similar to that of EGF treatment, namely induction of filopodia, whereas DexBSA treatment in combination with MβCD failed to induce formation of stress fibers (Supplementary Fig. 1a). To validate that these cytoskeletal changes are mediated by Cav1, we performed phalloidin staining using control, Cav1^KO^ and Cav1^OE^ keratinocytes. We found that overexpression of Cav1 resulted in an increase in stress fiber formation, even in the absence of stress fiber promoting stimuli, whereas Cav1 knockdown resulted in increased formation of filopodia (Supplementary Fig. 1b). Thus, these data suggest that GCs, via Cav1, induce reorganization of F-actin into more pronounced stress fibers.

### Induction of stress fibers by GCs is Cav1 dependent and is mediated by increase of RhoA and decrease of Cdc42 activities

**Fig. 3:**
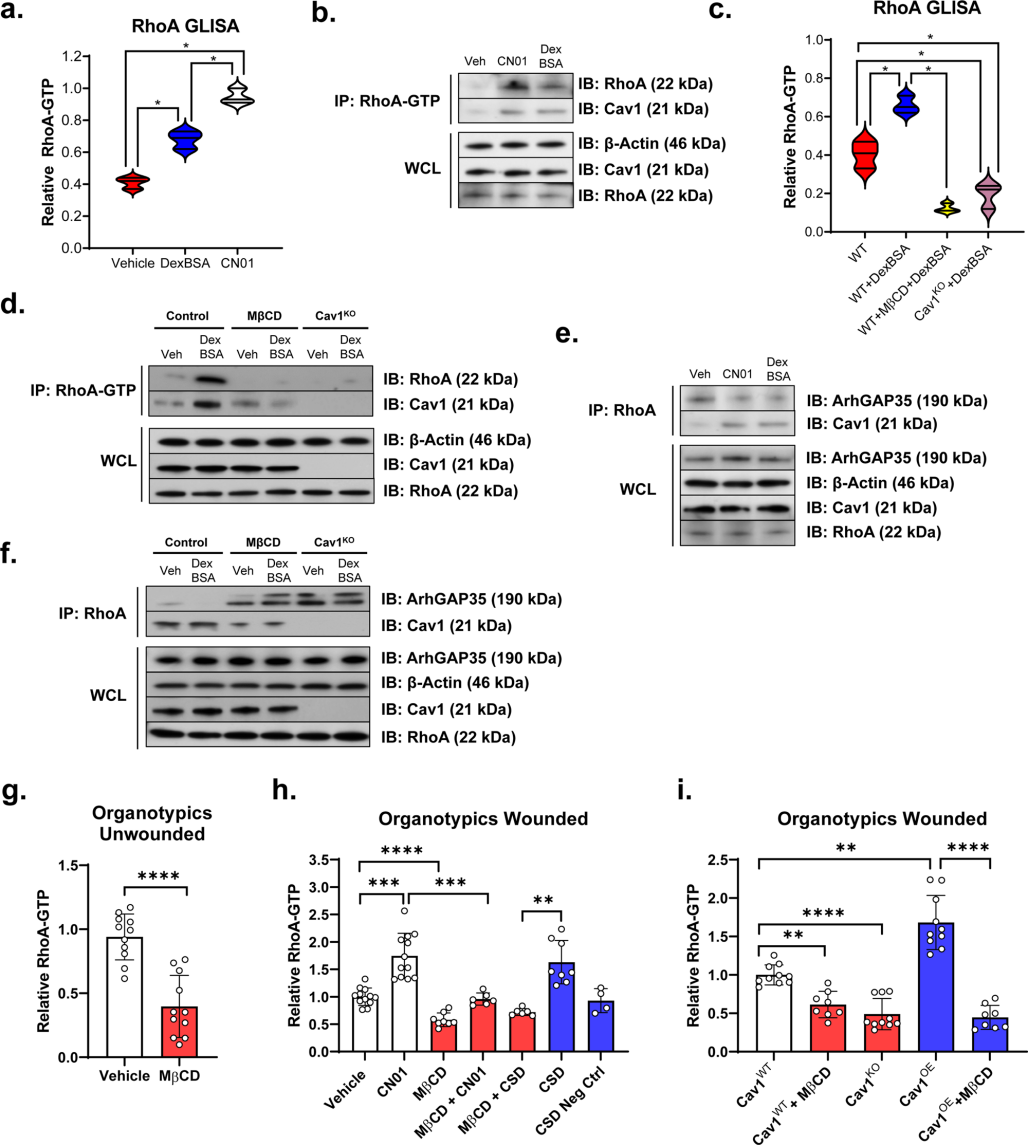
Glucocorticoids promote stress fiber induction by increasing RhoA activity in a Cav1-dependent manner. Primary human keratinocytes were treated with either DexBSA or a known RhoA activator CN01 (calpeptin), with RhoA activity measured by GLISA (**a**) and validated by pulldown experiments (**b**). Normal human keratinocyte cell lines and Cav1^KO^ keratinocytes were treated with DexBSA in presence/absence of MβCD, with RhoA activity measured by GLISA (**c**) and validated by pulldown experiments (**d**–**f**). Each GLISA experiment was carried out in triplicates from three independent experiments and statistical significance analyzed using paired Student’s *t* test, *p < 0.05. For pulldown experiments, both GTP- as well as total RhoA antibodies were used. Levels of active RhoA (**b**, **d**), relative to total RhoA (**e**, **f**), as well as their interaction with Cav1 and ArhGAP35 were determined by immunoblotting (**f**), with β-Actin serving as loading control. Primary human keratinocytes and fibroblasts were used to construct 3D human skin equivalent organotypics (**g**, **h**) cultures, which were treated in presence or absence of MβCD and caveolin scaffolding domain (CSD) peptide and subject to RhoA GLISA in order to determine RhoA-GTP status. CN01 served as a positive control for activation of RhoA. **i** Cav1^WT^, Cav1^KO^ and Cav1^OE^ keratinocyte cell lines in combination with normal primary human fibroblasts were used to construct 3D human skin equivalent organotypics cultures which were treated in presence or absence of MβCD, wounded and subjected to RhoA GLISA in order to determine RhoA-GTP status. Each GLISA experiment was carried out in triplicates from three independent experiments and statistical significance analyzed using paired Student’s *t* test, **p < 0.01, ***p < 0.001, ****p < 0.0001.

To gain mechanistic insights regarding the induction of stress fiber formation by cell migration inhibitor, GCs, we performed both RhoA G-LISA activation assays in combination with RhoA-total and RhoA-GTP immunoprecipitation experiments using human keratinocytes. We found that DexBSA triggered a statistically significant activation of RhoA, albeit to a lesser extent than that of calpeptin (CN01) (Fig. 3a). Interestingly, this activation of RhoA coincided with increased interaction of RhoA-GTP with Cav1, as evidenced by pull-down experiments (Fig. 3b), suggesting that Cav1 facilitates RhoA activation. Next, we sought to test if disruption of Cav1 (either by MβCD or in Cav1^KO^ cells) affects activation of RhoA. Both G-LISA (Fig. 3c) and immunoprecipitation experiments (Fig. 3d), demonstrated that GC-dependent activation of RhoA is dependent on Cav1. RhoA activation was significantly suppressed in Cav1^KO^ cells and MβCD-treated cells (Fig. 3c, d). As expected, the GC-mediated activation of RhoA also corresponded to a decreased interaction of RhoA with ArhGAP35 (Fig. 3e), which can be reversed by disruption of Cav1 (Cav1^KO^ cells or MβCD treatment) (Fig. 3f). Next, we utilized primary human keratinocytes and fibroblasts to generate 3D human skin equivalent organotypic cultures, wounded the organotypics in presence/absence of MβCD and a cell permeable caveolin scaffolding domain (CSD) peptide and assessed RhoA activation by G-LISA assays. We observed that MβCD treatment dampened activation of RhoA even in unwounded skin equivalents (Fig. 3g). Upon wounding, as expected, MβCD was able to reduce activation of RhoA, even in presence of potent RhoA activator CN01 or CSD peptide (Fig. 3h). We next utilized Cav1^KO^ and Cav1^OE^ keratinocyte cell lines in combination with primary human fibroblasts to develop organotypics and wounded them in presence/absence of MβCD. As expected, we demonstrated that MβCD treatment resulted in reduced activation of RhoA similar to that of Cav1^KO^ (Fig. 3i).

**Fig. 4:**
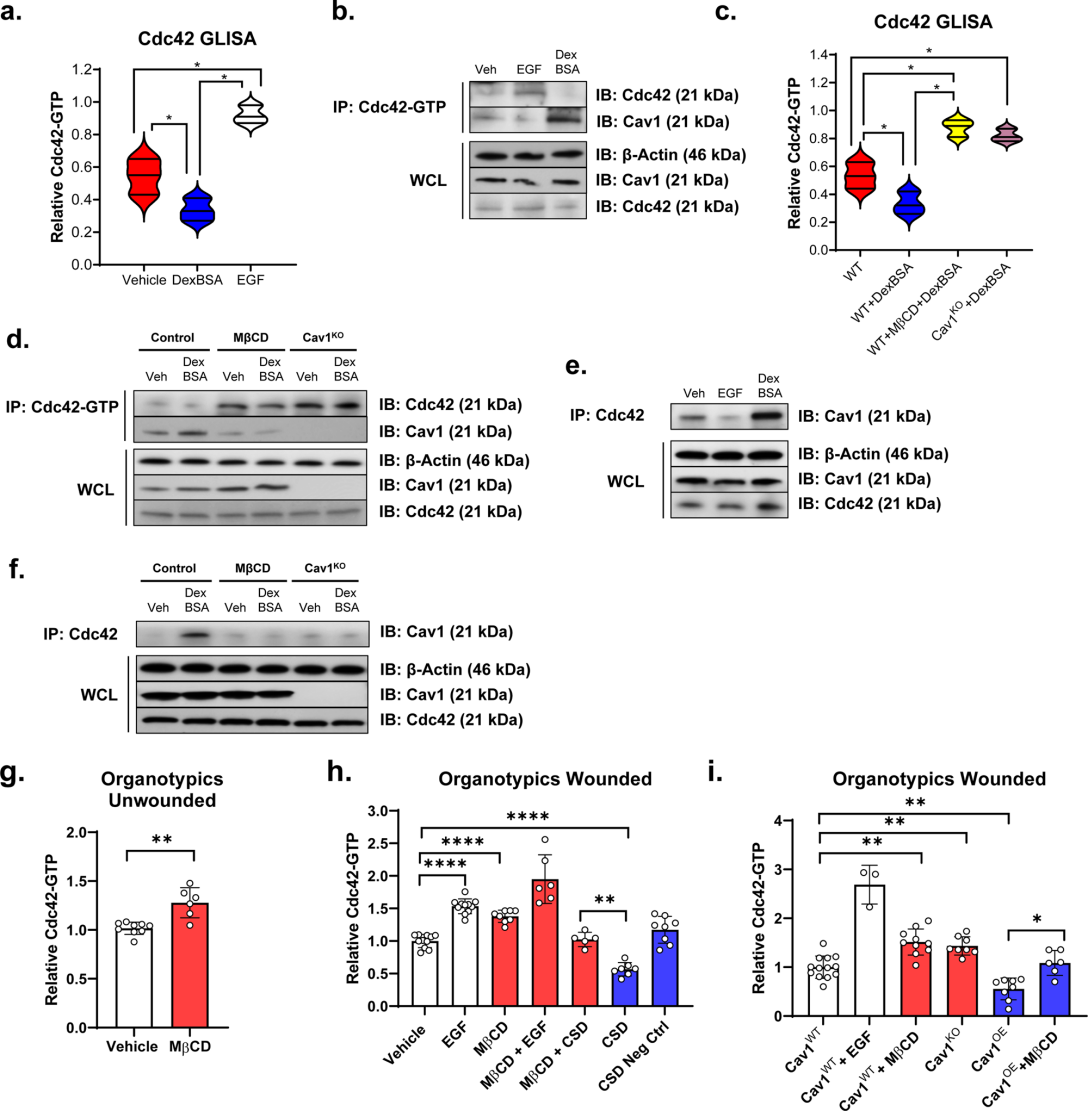
Glucocorticoids inhibit Cdc42 activity in a Cav1-dependent manner. Primary human keratinocytes were treated with either DexBSA or a known Cdc42 activator (EGF), with Cdc42 activity measured by GLISA (**a**) and validated by pulldown experiments (**b**). Normal human keratinocyte cell lines and Cav1^KO^ keratinocytes were treated with DexBSA in presence/absence of MβCD, with Cdc42 activity measured by GLISA (**c**) and validated by pulldown experiments (**d**–**f**). Cdc42 activity was measured by GLISA (**a**, **c**) and validated by pulldown experiments (**b**, **d**–**f**). Each GLISA experiment was carried out in triplicates from three independent experiments and statistical significance analyzed using paired Student’s *t* test, *p < 0.05. For pulldown experiments, both GTP- as well as total Cdc42 antibodies were used. Levels of active Cdc42 (**b**, **d**), relative to total Cdc42 (**e**, **f**), as well as their interaction with Cav1 were determined by immunoblotting, with β-Actin serving as loading control. Primary human keratinocytes and fibroblasts were used to construct 3D human skin equivalent organotypics (**g**, **h**) cultures, which were treated in presence or absence of MβCD and caveolin scaffolding domain (CSD) peptide and subject to Cdc42 GLISA in order to determine Cdc42-GTP status. EGF served as a positive control for activation of Cdc42. **i** Cav1^WT^, Cav1^KO^ and Cav1^OE^ keratinocyte cell lines in combination with normal primary human fibroblasts were used to construct 3D human skin equivalent organotypics cultures which were treated in presence or absence of MβCD, wounded and subjected to Cdc42 GLISA in order to determine Cdc42-GTP status. Each GLISA experiment was carried out in triplicates from three independent experiments and statistical significance analyzed using paired Student’s *t* test, **p < 0.01, ***p < 0.001, ****p < 0.0001.

RhoA and Cdc42 have been shown to exhibit known antagonistic behavior^31,32^ suggesting modulation of Cdc42 activity in the direction opposite of RhoA. We assayed activation of Cdc42 by GCs using Cdc42 G-LISA in combination with either Cdc42 total or Cdc42-GTP immunoprecipitation experiments. As expected, we observed that DexBSA diminished activation of Cdc42 (Fig. 4a). Interestingly though, we observed that DexBSA treatment also stimulated interaction of Cdc42 and Cav1, while EGF treatment disrupted this interaction (Fig. 4b), suggesting that Cav1 may lead to Cdc42 inactivation by sequestering Cdc42 upon GC treatment. Subsequently, we observed that disruption of Cav1 in keratinocytes (either in Cav1^KO^ cells or by MβCD treatment) reversed GC-mediated inactivation of Cdc42 and thus resulted in stimulation of Cdc42 activation (Fig. 4c–f). Similar to described above, when we utilized human skin equivalent organotypic cultures, MβCD treatment resulted in increased activation of Cdc42 in both unwounded and wounded organotypics, and was able to reverse the inhibition of Cdc42 observed by administration of CSD (Fig. 4g, h). Furthermore, organotypics made with Cav1^OE^ exhibited diminished Cdc42 activity, whereas Cav1^KO^ organotypics exhibited elevated Cdc42 activity, similar to that of MβCD treatment (Fig. 4i). Together, these data suggest that Cav1 may affect cell migration through reorganization of actin-cytoskeleton by promoting induction of stress fibers via activation of RhoA, while at the same time inhibiting activation of Cdc42.

### Cav1 knockdown accelerates epithelialization in vivo

**Fig. 5:**
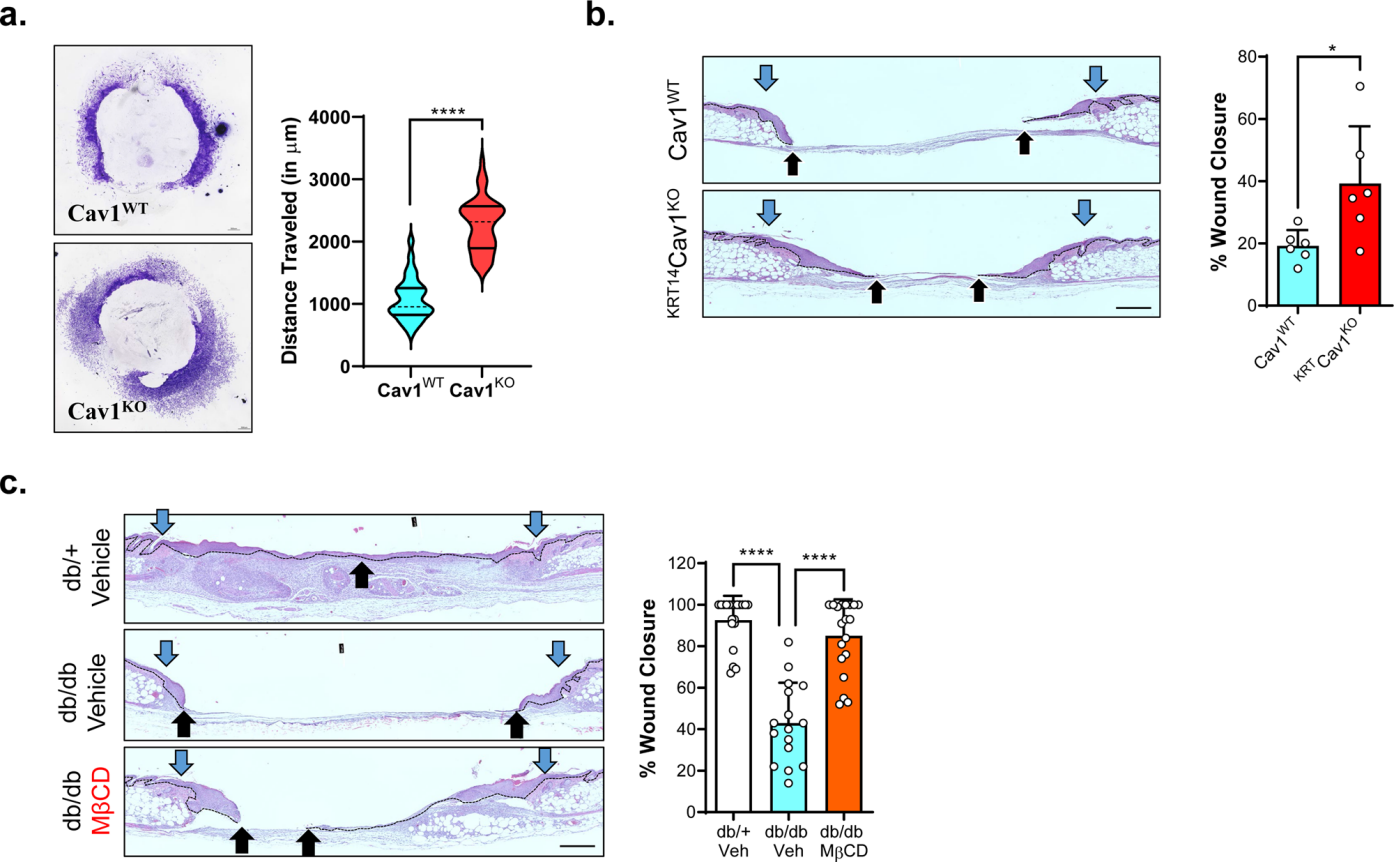
Cav1 disruption accelerates wound closure in vivo. **a** Re-epithelialization potential of in Cav1^WT^ and constitutive/global Cav1^KO^ mice was assessed by epithelial explant outgrowths emerging from the mouse skin explants. Explant outgrowth was quantified by measuring the total distance of cells traveled measured from the concentric tissue explant to the outermost edge as well as by spectrophotometry of crystal violet solubilization of cells protruding from the explant outgrowth. *n* = 20 independent biological specimens, *p < 0.0001 unpaired *t*-test. **b** Rates of re-epithelialization were validated using splinted model of wound closure using mice expressing endogenous levels of Cav1 (Cav1^WT^), Cav1 global knockout mice (Cav1^KO^) and tamoxifen-inducible keratinocyte specific Cav1 knockout mice (^KRT14^Cav1^KO^) with blue arrows corresponding to wound edge and black arrows corresponding to migrating epithelial tongue; scale bar = 500 µm. *n* = 6, *p < 0.05, one-way ANOVA using Tukey correction for multiple comparisons. **c** Rates of re-epithelialization were assayed in chronic wound model using db/db mice treated with ±MβCD with blue arrows corresponding to wound edge and black arrows corresponding to migrating epithelial tongue. *n* = 20, ***p < 0.001, one-way ANOVA using Tukey correction for multiple comparisons.

To determine the functional relevance of Cav1 knockdown in skin re-epithelialization, we first analyzed the potential of keratinocyte migration using the well-established skin explant ex vivo model^33,34^. We observed that skin explants from Cav1^KO^ mice (constitutive/global Cav1 knockout mice) show significantly higher levels of keratinocyte outgrowth quantified both by the overall increase in the number of protruding cells from the explant and the distance they traveled (Fig. 5a). We validated that the migrating cells were indeed activated (K6a^+^/K17^+^) non-differentiating (K10^−^), basal (K14^+^/K15^−^) keratinocytes (Supplementary Fig. 3a).

Subsequently, we then compared rates of re-epithelialization in Cav1^WT^ (C57BL6/J) and ^KRT14^Cav1^KO^ (tamoxifen-inducible keratinocyte specific Cav1 knockout mice under the K14 promoter) using a splinted model of wound healing^35^. Indeed, ^KRT14^Cav1^KO^ mice exhibited faster re-epithelialization in comparison to the their Cav1^WT^ counterparts (Fig. 5b). Next, we tested functionally how Cav1 disruption affects re-epithelialization in a healing delayed splinted wound model using db/db mice. Skin of db/+ or db/db mice was topically treated either with 1% MβCD (w/v) or vehicle control 48 h prior to wounding, and wound re-epithelization was assessed by histomorphometry. As expected, topical MβCD treatment reversed the delayed re-epithelialization commonly observed in db/db mice (Fig. 5c). Interestingly, Cav1 and Cavin1 seem to exhibit differential expression/localization patterns during acute wound healing, where Cav1 exhibits a spatiotemporal downregulation in basal keratinocytes (Supplementary Fig. 4a), whereas Cavin1 expression/localization does not seem to change during acute wound healing (Supplementary Fig. 5a). Thus, the observed effects on physiological cutaneous wound closure seem to be Cav1 specific. However, we observed that diabetic db/db mice (which exhibited retarded rates of wound closure), also exhibited upregulation of both Cav1 and Cavin1 expression as well as a change in localization to both basal and suprabasal layers of the epidermis (Supplementary Figs. 4b and 5b). Thus, during pathophysiological wound closure, both Cav1 and Cavin1 may be involved in obstructing directional cell migration and successful wound closure. MβCD treated db/db murine skin however reversed the impeded wound closure observed in the db/db mice, possibly due to modulation of caveolae via cholesterol depletion, as both Cav1 and Cavin1 were observed localized to the cytoplasm of epidermal keratinocytes. Together these data confirm that targeted downregulation of Cav1 in keratinocytes accelerates wound closure under both physiological and pathophysiological in vivo and ex vivo mouse and human models, revealing new mechanisms that inhibit wound closure in patients.

## Discussion

**Fig. 6:**
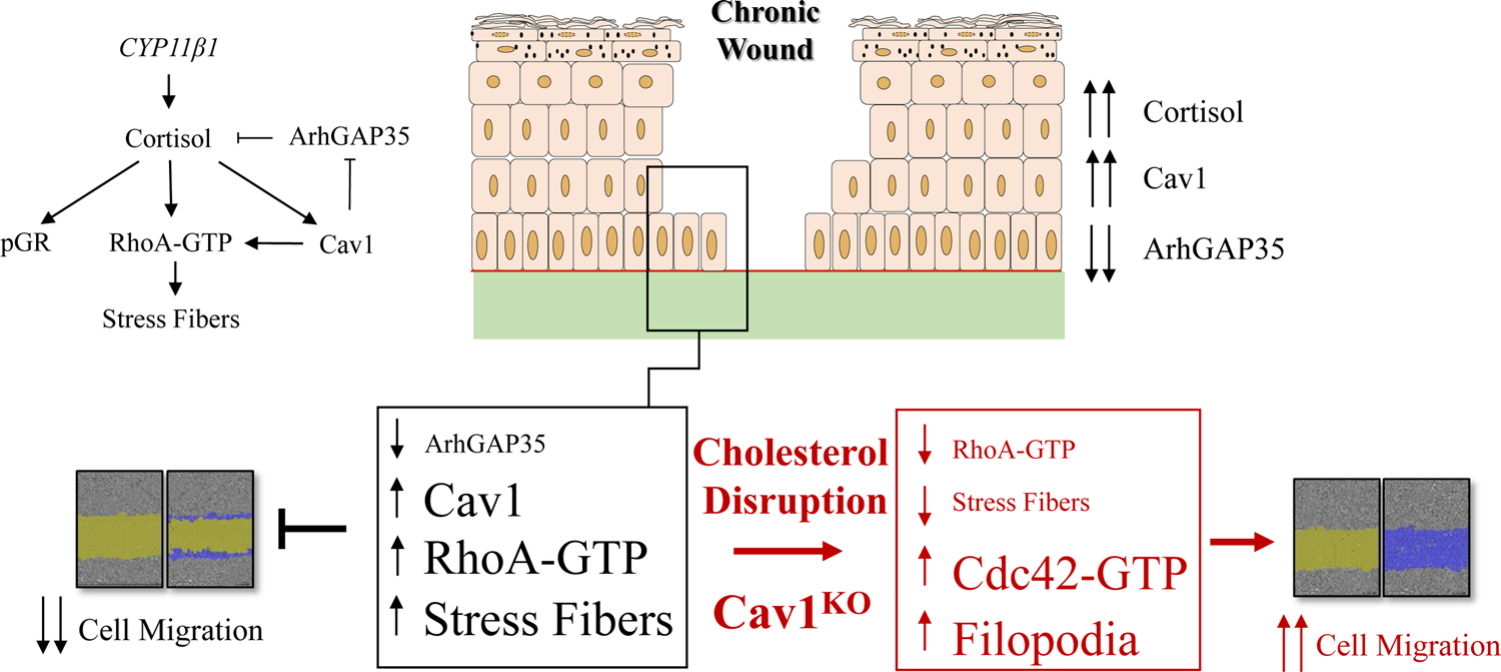
Proposed mechanism of action. Increased cortisol synthesis in DFUs which binds to and activates glucocorticoid receptor leads to increased levels of active cortisol, which subsequently leads to downregulation of GR repressor ArhGAP35 as well as an upregulation of Cav1. Downregulation of ArhGAP35 stimulates activation of RhoA and induction of stress fibers to inhibit directional keratinocyte migration and wound closure. Perturbation of caveolae (by MβCD) or knockout of Cav1, reverses corticosteroid-mediated induction of stress fibers and inhibition of directional cell migration and may be used as a potential therapeutic target to promote wound re-epithelialization of non-healing chronic wounds.

Inhibition of keratinocyte migration clinically observed in both types of chronic wounds (DFUs and VLUs)^11^ is supported by genomic analyses that identified complex deregulation of genes responsible for cytoskeletal organization and cell movement. In addition, we provide evidence for a novel mechanism by which Cav1 orchestrates cytoskeletal compartmentalization that regulates keratinocyte migration and subsequent wound closure in a glucocorticoid-dependent manner. GCs are known inhibitors of keratinocyte migration and wound closure^12,24,36^. Here we show that GCs act as agonists of Cav1 and demonstrate that excess cortisol production (found in chronic wound patients) promotes expression of Cav1 and its upregulation in chronic wounds^12,15^. We show that this, in turn, leads to a suppression of a glucocorticoid receptor repressor GRLF1 (aka p190RhoGAP and ARHGAP35), which also functions as a GTPase activating protein for RhoA. Consequently, this leads to increased activation of RhoA and formation of stress fibers, and a concomitant inhibition of Cdc42 activation, all in a Cav1-dependent manner. Thus, Cav1 disruption, either genetically (using keratinocyte specific Cav1 knockout mice) or pharmacologically (using MβCD), can accelerate physiological wound closure as well as reverse the delayed wound closure observed in db/db mice (Fig. 6).

It has been well established that activity of Cdc42, Rac1 and RhoA must be spatiotemporally regulated in order for the cell to achieve directional mobility, starting with the filopodial protrusion (regulated by Cdc42), followed by lamellipodial extension (regulated by Rac) and then by stress fiber contraction (Rho). Perturbation of any of these intricate signaling pathways results in random cellular movement or no movement at all. Interestingly, it has been demonstrated that in rabbit corneas, significant increase in Cdc42 expression that occurs 2–4 days after the injury coincided with wound closure, which by day 8 returned to near basal levels. Furthermore, silencing of Cdc42 expression in cultures caused inhibition of wound closure as a result of 60–75% decrease in epithelial migration and growth^16,37^. Moreover, we have recently shown that topical mevastatin can promote directional cell migration by targeting Cav1/EGF pathway resulting in sustained activation of Rac1^15^. Thus, multiple lines of evidence point to tight regulation of Rho/Rac/Cdc42, not just in terms of expression, but also as in terms of activity as demonstrated in the current manuscript, in order to achieve desired healing outcomes.

Although our observations which point to Cav1 promoting RhoA signaling and antagonizing Cdc42 signaling are consistent with what is currently accepted in the literature^15,31,38–43^, the role of Cav1 in cell migration, however, has been controversial as some studies suggest that it promotes^14,44,45^ while others suggest it inhibits^12,15,46–49^ cell migration. These paradoxical observations can be attributed to cell specific effects, since Cav1 can bind to and associate with various signaling molecules, meaning that overall potential of Cav1 to promote or inhibit migration may depend on the availability of numerous signaling molecules from cell receptors to actin-based cytoskeletal proteins. Similar to the data presented here on cutaneous wounds, Cav1 expression levels are inversely related to corneal wound healing capacity and Cav1 ablation results in faster re-epithelialization of the cornea^49,50^.

The diverging findings on the role of Cav1 extend to wound healing observations from contributing to inhibition^12,15,50^, to promoting wound closure^14,51^. The apparent difference can be attributed to use of different models and assessment methods to study wound closure, diversity of wound types (i.e., burns vs chronic wounds) and lack of models that recapitulate the human condition (reviewed in^52^). Interestingly, Grande-Garcia et al.^14^, demonstrated that Cav1 global knockout mice exhibit delayed wound closure in comparison to Cav1^WT^ littermates, due to ineffective persistence of fibroblast migration in Cav1^KO^ mice. Since numerous studies have shown that Cav1 plays a role in mechanotransduction^53–57^, controlling for wound contraction would be necessary. To reconcile these seemingly controversial findings, we developed a keratinocyte specific Cav1 knockout mouse model (^KRT14^Cav1^KO^) that focuses on the role of Cav1 in epidermis and used a splinted wound model designed to test wound closure by epithelialization^35^. Using the inducible ^KRT14^Cav1^KO^ model, we confirmed that targeted downregulation of Cav1 in epidermis accelerates re-epithelialization and wound closure. Similarly, topical application of caveolae disrupting agent MβCD in diabetic wounds in vivo also promotes wound closure, which is further supported by data from patients and genetically modified organotypic skin cultures. Induction of Cav1 is found in patients with non-healing wounds and human ex vivo organotypic skin generated from keratinocytes that overexpress Cav1 shows delayed closure^12^. Thus, multiple lines of in vivo and ex vivo evidence supports the conclusion that downregulation of Cav1 facilitates keratinocyte migration and wound closure, whereas induction of Cav1 inhibits it. Although MβCD is commonly utilized to extract cholesterol from cell membranes and not to specifically disrupt Cav1, it appears that disruption of caveolae (by either cholesterol depletion or cavin-1 silencing) leads to its destabilization and degradation (at least in certain cell types)^58,59^. Along these lines, we have also recently demonstrated that disrupting cholesterol synthesis using HMG-CoA reductase inhibitor (mevastatin) can diminish protein levels of Cav1 in primary human keratinocytes and in human skin samples^15^. Thus, our observations that cyclodextrins can also disrupt Cav1 levels should not be that surprising.

Interestingly, recent findings from Robert Parton’s group have demonstrated that caveolins possess essential cellular functions independent of caveolae as determined by caveolin expressing cells that do not develop caveolae^60^. In this sense, non-caveolar caveolins may interact with numerous proteins (and lipids) that may be excluded from caveolae by oligomerizing into scaffolds of lipid-ordered domains, and it is speculated that this may be one of the ways by which they control actin-cytoskeleton organization outside of the cell membrane^61^. Our observation, that acute wounds exhibit differential expression/localization of Cav1 and cavin1, where Cav1 exhibits a spatiotemporal downregulation in basal keratinocytes (while cavin1 expression remains constant) (Supplementary Figs. 4 and 5), further point to non-caveolar functions of Cav1 in regulation of actin cytoskeleton and subsequent cell migration/wound closure. It is possible however, that during pathophysiological wound closure (as observed in db/db mice), upregulation of both Cav1 and cavin1 may lead to deregulation of both signaling events at the level of caveolae, as well as intracellular trafficking at the level of endosomes. Since it appears that many of the structural components of caveolae are upregulated in chronic wounds (Cav1, cavin2, cavin3, cavin4) (Fig. 1b), it is plausible to assume that chronic wounds may exhibit elevated number of caveolae, which may be involved in numerous cellular processes that affect wound closure, including transcytosis of various intracellular cargo. Therefore, the overall contribution of caveolae and lipid rafts to successful wound closure warrants further investigation. However, seeing that Cav1 knockdown, either in vitro by CRISPR-Cas9-mediated gene knockdown or in vivo by utilizing tamoxifen-inducible ^KRT14^Cav1^KO^ mice, accelerates directional cell migration and subsequent wound closure, together these data argue for a Cav1-specific effect, rather than being a secondary effect resulting from disruption of caveolar transport.

In conclusion, we identified a previously unreported mechanism by which Cav1 inhibits keratinocyte migration and wound closure, which is clinically observed in non-healing chronic wounds, such as DFUs and VLUs. This mechanism shows that Cav1 regulates activity and localization of Rho GTPases and orchestrates re-organization of cytoskeletal machinery in a glucocorticoid-dependent manner, which can be reversed by disruption of Cav1 in multiple models of wound closure in vivo and ex vivo. In addition to Cav1, we identified aberrant expression of actin-related cytoskeletal genes in both VLUs and DFUs, which emphasizes the importance of membrane organization as a key mediator of keratinocyte migration and wound closure. Taken together, our findings provide a new therapeutic approach to stimulate wound healing via disruption of Cav1.

## Materials and methods

### Antibodies and reagents

Antibodies for immunoblots were used as follows: Cav1 (1:2000, Cell Signaling, #3267); ArhGAP35 (1:1000, Sigma, #HPA055184); RhoA (NewEast Biosciences, 1:500, #21017); Cdc42 (NewEast Biosciences, 1:250, #21010); β-Actin (1:10,000, Sigma, A5441); anti-rabbit IgG HRP (1:1000, Cell Signaling, #7074); anti-mouse IgG HRP (1:1000, Cell Signaling, #7076). Antibodies for co-immunoprecipitation were as follows: RhoA-GTP (NewEast Biosciences, #26904); RhoA-total (NewEast Biosciences, #26007); Cdc42-GTP (NewEast Biosciences, #26905); Cdc42-total (NewEast Biosciences, #26008). Antibodies and reagents for immunohistochemistry and immunofluorescence were ArhGAP35 (1:100, Sigma, #HPA056470); Cav1 (1:200, Sigma, #HPA049326); Cavin1 (1:750, Cell Signaling #69036); K6a (1:100, BioLegend, #905701); K17 (1:0, Santa Cruz, #sc393091); K10 (1:200, Sigma, #HPA012014); K14 (1:500, BioLegend, #905301); K15 (1:100, Sigma, #SAB4501658); Phalloidin-rhodamine (100 nM, Cytoskeleton, #PHDR1).

### Cell culture

Primary adult human foreskin epidermal keratinocytes were grown as previously described^12^. Normal keratinocytes were isolated from skin of individuals undergoing reduction surgeries, whereas DFU keratinocytes were isolated from individuals undergoing standard of care debridement at University of Miami Wound Clinic and grown in a serum free keratinocyte growth medium (keratinocyte SFM; Gibco #10724-011, Grand Island, NY, USA) supplemented with 0.2 ng/mL epidermal growth factor, 25 µg/mL bovine pituitary extract, and 1% penicillin/streptomycin at 37 °C in 5% CO_2_. Third passage cells were used at 60~70% confluency. Keratinocytes, grown on 60 mm plates (Greiner Bio One #628160, Frickenhausen, Germany), were then starved overnight with custom basal keratinocyte medium (Basal KSFM without phenol red, T3, hydrocortisone, insulin and L-Glutamine (Gibco, Grand Island, NY, USA) prior to treatment with ±1% (w/v) MβCD (Sigma #F4767, St Louis, MS, USA) or for 30 min and then stimulated with ±DexBSA (Steraloids, Newport, RI, USA) at appropriate concentrations. The cells were harvested with a cell scraper according to the allotted time for each experiment.

### Animals

All animal care and use procedures were approved by the University of Miami Institutional Animal Care and Use Committee (protocols #18-053 and #19-197). Global Cav1^KO^ (B6.Cg-Cav1^tmMls^/J; stock #007083), K14cre-ER^tam^ (Tg(KRT14-Cre/ERT)20Efr/J; stock #005107) Krt14-Cav1^KO^, db/+ or db/db (Lepr^db^; stock #000697) and BL/6 (C57BL/6J; stock #000664) were all purchased from The Jackson Laboratory. Mice carrying a Cav1 conditional knockout allele that is flanked by loxP sites (Cav1-flox/flox^62,63^, generous gift from Timothy Thompson) were crossed with transgenic mice expressing tissue-specific Cre recombinase fused to a mutated human estrogen receptor (ER) in basal keratinocytes driven by the keratin 14 promoter (tamoxifen-inducible K14cre-ER^tam^) in order to obtain keratinocyte specific Cav1-deleted mice. Cre nuclear translocation was induced by topical application of 4-OH tamoxifen (1 mg for 3 consecutive days), in order to obtain tissue-specific Cav1 knockout mice (^KRT14^Cav1^KO^).

### Human skin specimens

Control healthy human skin specimens were obtained as discarded tissue from reduction surgery procedures in accordance to institutional approvals as previously described^12^. Specifically, protocol to obtain unidentified, discarded human skin specimens from reduction surgery was submitted to University of Miami Human Subject Research Office (HSRO). Upon review conducted by University of Miami Institutional Review Board (IRB) it was determined that such protocol does not constitute Human Subject Research. As such, this project was not subject to IRB review under 45 CFR46.101.2. Chronic wound samples were obtained from consenting patients receiving standard care at the University Of Miami Hospital. These protocols were approved by the Institutional Review Board (IRB protocols #20150912; #20150222; #20090709, #27085). Ulcers did not exhibit any clinical signs of infection. Chronic wound tissue was either stored in RNALater(Applied Biosystems, Carlsbad, CA) for subsequent RNA isolation, snap frozen (protein isolation), or fixed in formalin (paraffin embedding). All patient demographics were included in Supplementary Table 1.

### Ex vivo human wound model

Human skin specimens from reduction surgery were used to generate acute wounds as previously described^64,65^. Briefly, a 3 mm biopsy punch was used to create acute wounds (*n* = 9 per treatment) which were treated daily with or without 0.1 µM DexBSA or 2% (w/v) MβCD or combination for 5 days. Ex vivo acute wound specimens were paraffin embedded and rate of healing was analyzed for epithelialization by histology assessment using a Keyence BZ-X700 microscope.

### Explant outgrowth

Explant outgrowth procedure was performed as previously described^33^. Two-day old BL6 and Cav1^KO^ were used to create 4 mm explants, which were then placed onto a 24-well dish, allowed to attach and then supplemented with 200 ul of media. Explants were incubated (37 °C, 5% CO_2_) for 8 days and imaged daily in order to quantify outgrowth. After the completion of the quantification, cells were stained with crystal violet, dissolved in 10% acetic acid and absorbance measured at 590 nm.

### In vivo wounding

All animal care and use procedures were approved by the University of Miami Institutional Animal Care and Use Committee. Under anesthesia, hair on the dorsal skin of female mice global Cav1^KO^ (B6.Cg-Cav1^tmMls^/J; stock #007083; The Jackson Laboratory), ^KRT14^Cav1^KO^, db/+ or db/db (Lepr^db^; stock 000697; The Jackson Laboratory) eight weeks of age, was removed by clipping and application of chemical depilatory cream (Veet^TM^) for 30 s, after which the skin was cleaned with water and antiseptics. Mice were treated with 1% MβCD (w/v) dissolved in ice-cold F127 Pluronic gel, with Pluronic gel alone serving as vehicle control. Forty-eight hours later, two-5mm full-thickness excisional wounds were created in the dorsal skin on both sides of the midline, after which 10 mm donut shaped silicone splint was sutured using Ethilon 6-0 sutures and covered by Tegaderm as previously established by Gurtner group^35^. Wound tissue was collected on either day 3, 5 or 7 post-wounding, fixed in 10% formalin, processed, and embedded in paraffin. Paraffin sections (5 µm) were stained with hematoxylin and eosin for histological analysis, and immunohistochemistry experiments.

### Immunohistochemistry

Formalin fixed/paraffin embedded tissue was cut at 5-7μm sections using a microtome. Slides containing sections were deparaffinized with xylene (EMD, Gibbstown, NJ, USA), and rehydrated in graded ethanol. For immunoperoxidase experiments, endogenous peroxidase activity was quenched with 0.3% H_2_O_2_ in methanol and washed with distilled water. The slides were then incubated in sodium citrate buffer (10 mM sodium citrate, 0.05% Tween-20, pH 6.0) for 30 min at 95 °C for antigen retrieval, allowed to cool down, and then treated with Background punisher (MACH1 kit, Biocare Medical, Concord, CA, USA). Antibodies were diluted in 2% normal goat serum (Sigma-Aldrich, St. Louis, MO, USA) in PBST (PBS, 0.1% Tween-20) and applied to the samples for overnight incubation at 4 °C. The detection and chromogenic reaction were carried out using the MACH 1 Universal HRP-Polymer Detection system (Biocare Medical, Concord, CA, USA) and following manufacturer’s instructions. Slides were counterstained using Harris hematoxylin (Leica Microsystems, Wetzlar, Germany), and then dehydrated in graded ethanol and xylene. Sections were imaged and analyzed using a Keyence BZ-X700 microscope. For immunofluorescence experiments, image-iT FX signal enhancer (Molecular Probes, Cat. No. I36933) was applied to rehydrated tissue specimens for 30 min followed by incubation 5% bovine serum albumin in PBST, prior to incubation with appropriate antibody overnight. The following day, sections were then counterstained with AlexaFluor 488/594 coupled secondary antibodies for 1 h prior to mounting in ProLong^TM^ Gold Antifade mounting medium with DAPI (ThermoFisher Scientific, Inc). Sections were imaged and analyzed using a Keyence BZ-X700 microscope.

### Quantitative PCR

RNA isolation and purification were performed as previously described^24^. 1.0 µg of total RNA from HEK was reverse transcribed using a qScript cDNA kit (QuantaBio, Beverly, MA) and real time PCR was performed in triplicates using the Bio-Rad CFX Connect thermal cycler and detection system and a PerfeCTa SYBR Green Supermix (QuantaBio, Beverly, MA). Relative expression was normalized for levels of Arpc2. Primer sequences can be found in Supplementary Table 2. Statistical comparisons of expression levels from, chronic wound *vs* normal skin was performed using a paired Student’s *t* test.

### RhoA and Cdc42 activation experiments

Activation of RhoA and Cdc42 was assesses using GLISA activation experiments and validated using affinity chromatography co-immunoprecipitation experiments. Where indicated, cells were treated for 24 h with 3 µM cell permeable Caveolin Scaffolding Domain peptide (Millipore, #219842) or scrambled version of Cav1 Scaffolding Domain Negative Control Peptide (Millipore, #219483), as previously described^66–69^. *GLISA Activation*: GLISA activation assays were performed according to manufacturer’s instructions (Cytoskeleton Inc., RhoA: #BK124; Cdc42: #BK127). Briefly, cells were grown to 60% confluency and serum starved overnight prior to assessment of Cdc42/RhoA activity. The following day they were stimulated for either 30 s (for Cdc42 activity) or 20 min (for RhoA activity) in presence/absence of 1% MβCD (w/v) prior to washing once in ice-cold PBS, lysing in ice-cold lysis buffer (Cytoskeleton, Inc), clarified by centrifugation and snap frozen in order to preserve GTP status of Cdc42/RhoA. 25 ul of 1 mg/ml triplicates for each condition were used in each GLISA experiment and signal was read by measuring absorbance at 490 nm using a microplate spectrophotometer. Each experiment was repeated three times and statistical comparison of activation levels was performed using a paired Student’s *t* test. *Co-Immunoprecipitation Experiments*: Co-immunoprecipitation protocol was followed as previously described^70^ with minor modifications using the Pierce Classic IP kit (Thermo Scientific #26149, Rockford, IL, USA). Briefly, lysis buffer (25 mM Tris-HCl, 150 mM NaCl, 1 mM EDTA, 1% NP-40, 5% glycerol, pH 7.4) + Protease/Phosphatase Inhibitor Cocktail were added directly to the keratinocyte monolayer seeded on 60 mm plates and incubated on ice for 10 min, after which cell debris was pelleted by centrifugation at 13,000 × *g* for 10 min at 4 °C. Pre-cleared cell lysate (400 µg) was incubated with 1 µg of antibody (NewEast Biosciences, Inc.) immobilized on AminoLink Plus Coupling resin overnight at 4 °C, after which the resin was washed extensively with lysis buffer, eluted and solubilized in SDS sample loading buffer.

### Western blotting

To prepare whole cell lysates, keratinocytes were washed twice with ice-cold PBS and lysed in ice-cold lysis buffer (20 mM Tris-HCl pH 7.5, 150 mM NaCl, 1% Triton X-100)^71^. The lysates were clarified by centrifugation, and protein concentrations were determined using the BCA Protein Assay Reagent Kit (Thermo Scientific). Proteins were resolved by 4–20% Criterion TGX pre-cast gels (Bio-Rad), transferred to polyvinylidene difluoride membranes (Thermo Scientific) and placed in blocking buffer for 1 h (TBS, 0.1% Tween20, 5% BSA) then probed with indicated antibodies overnight. All uncropped images of resulting western blots were included in Supplementary Fig. 7.

### CRISPR/Cas9 Cav1 knockout

Caveolin-1 CRISPR/Cas9 KO and caveolin-1 HDR plasmids (Santa Cruz Biotechnology, Dallas, TX) were used to generate Cav1 human keratinocyte knockout cells as previously described^12^. Briefly, 2 μg of Cav1-CRISPR/Cas9 and Cav1-HDR plasmids were combined with antibiotic/serum-free media containing FugeneHD (Promega, Madison, WI) transfection reagent and incubated for 48 h at 37 °C prior to proceeding with puromycin selection for 5 days.

### 3D organotypic skin equivalent cultures

Fibroblasts-collagen matrix was constructed using adult human primary dermal fibroblasts and type I collagen from bovine origin (Advanced BioMatrix, Inc., CA)^72–74^. Briefly, 1 ml of acellular collagen matrix was poured into PET membrane inserts (Greiner Bio-One North America Inc., NC) placed in 6 well plates (Corning Inc., NY, USA) and allowed to gellify at 37 °C for 20 min. 2.5 ml of cellular matrix containing the fibroblasts was added on top of the gellified acellular matrix and incubated at 37 °C for 30 min before adding organotypic culture medium (FAD medium supplemented with 10% FBS, 10^–10^ M cholera toxin, 0.4 µg/ml hydrocortisone per ml and 50 µg/ml L-ascorbic acid). The submerged matrices were incubated overnight at 37 °C. The next day, the media was removed from the matrices and 10^6^ HaCaT (normal, Cav1^KO^ or Cav1^OE^) cells per insert were added on top of each matrix. The cells were allowed to attach at 37 °C for 30–60 min, then the cultures were submerged into organotypic culture medium supplemented with 2 ng/ml EGF and incubated over night at 37 °C before raising them to air-liquid interface. Medium was replaced every 2 days for 20 days. Wounds were created by as described above. The wounds were treated daily for 2 days, collected and used for G-LISA experiments as depicted above.

### Statistics and reproducibility

The numbers of normal skin and chronic wound samples, organotypic 3D skin equivalents, normal, diabetic or Cav1 knockout mice, G-LISA replicates, or replicates of cell samples in cultured cell experiments, were indicated in each figure. All data were presented as mean ± SD. Student’s *t* test and either 1-way or 2-way ANOVA were performed and indicated in each of the figures. In all descriptions, *p < 0.05, **p < 0.01, ***p < 0.001 and ****p < 0.0001. Cultured cell-related experiments were repeated at least three times and similar results were obtained.

### Reporting summary

Further information on research design is available in the Nature Research Reporting Summary linked to this article.

## Supplementary information

Supplementary Information

Description of Supplementary Files

Supplementary Data 1

Reporting Summary

## Data Availability

All data associated with this study are available in the main text or the supplementary materials (Supplementary Data 1).
