# Supplementary material for: Glucocorticoid-mediated induction of caveolin-1 disrupts cytoskeletal organization, inhibits cell migration and re-epithelialization of non-healing wounds
Source: Commun Biol. 2021 Jun 18;4:757. doi: 10.1038/s42003-021-02298-5 (PMC8213848; doi:10.1038/s42003-021-02298-5)
Supplement: Supplementary file 1 — Supplementary Information [file 42003_2021_2298_MOESM1_ESM.pdf]

Skin ID	Age	Sex	Race	Type of specimen	Location
DFU_01a	69	M	W	DFU Wound	Plantar foot
DFU_01b				Adjacent Normal Skin	Plantar foot
DFU_02a	52	M	H	DFU Wound	Plantar midfoot
DFU_02b				Adjacent Normal Skin	Plantar midfoot
DFU_03a	71	M	H	DFU Wound	Plantar medial forefoot
DFU_03b				Adjacent Normal Skin	Plantar medial forefoot
DFU_04a	57	M	AA	DFU Wound	Plantar heel
DFU_04b				Adjacent Normal Skin	Plantar heel
NFS_01	83	F	H	Normal Foot Skin	Plantar metatarsal
NFS_02	61	M	W	Normal Foot Skin	Plantar big toe
NFS_03	43	F	AA	Normal Foot Skin	Plantar foot
NFS_04	63	M	W	Normal Foot Skin	Plantar 2nd toe
dDFU_01	60	M	H	Discarded DFU	Calcaneus
dDFU_02	78	M	H	Discarded DFU	Plantar foot
dDFU_04	64	M	H	Discarded DFU	Anterior plantar foot
dDFU_05	69	M	W	Discarded DFU	Plantar foot

Skin ID	Age	Sex	Race	Type of specimen
NDS_01	59	M	W	Normal dorsal foot skin
NDS_02	62	F	W	Normal dorsal foot skin
NDS_03	65	F	W	Normal dorsal foot skin
NDS_04	67	F	W	Normal dorsal foot skin
VLU_01	58	F	H	VLU
VLU_02	83	F	AA	VLU
VLU_03	55	M	W	VLU
VLU_04	60	M	W	VLU
VLU_05	61	M	W	VLU
VLU_06	61	M	AA	VLU
VLU_07	72	M	W	VLU
VLU_08	54	M	W	VLU
VLU_09	38	F	W	VLU
VLU_10	57	F	H	VLU
VLU_11	57	F	H	VLU
VLU_12	57	F	H	VLU
VLU_13	51	F	H	VLU

Supplementary Table 1. Patient demographics of patient biopsies used in this study. Plantar normal foot skin (NFS), diabetic foot ulcer (DFU), normal dorsal foot skin (NDS) and venous leg ulcer (VLU).

	Forward Primer	Reverse Primer
**ArhGAP15**	GATACTTCCGTGGAAACACTGAA	ACATCGGTGAGGATCATGGATTT
**ArhGAP24**	GAACCGTCTGGCTCCGATG	TGGCAGTCGAAAGAGACCCT
**ArhGAP29**	CCTTATGGGAGATGTAGGCAATG	AGCTCGATAGAGTCAGTGTTCT
**ArhGAP35**	CCCGAGTGCTGACGAGTTTC	GCGGCTAACTTCTCCCCAG
**ArhGEF06**	TCCTCGCTGAAAAATGGGGTA	CTTGGAGGGTTGCACATCCT
**ArhGEF09**	CCTAGAGCACCAAGATGGATTCT	GCCAACTGTAAGGGATACTTGC
**ArhGEF11**	CGCTGTGTCATTATCCAAAAGGA	CATGGTGCCGTTGACTTTGA
**Arpc2**	TCCGGGACTACCTGCACTAC	GGTTCAGCACCTTGAGGAAG
**Cav1**	GCGACCCTAAACACCTCAAC	ATGCCGTCAAAACTGTGTGTC
**Cavin1**	GAGGACCCCACGCTCTATATT	CCCCGATGATTTTGTCCAGGA
**Cavin2**	AGCAGCGACAGATCAGTTTGG	TATCCATGCGCTCTTTGACCG
**Cavin3**	CACGTTCTGCTCTTCAAGGAG	TGTACCTTCTGCAATCCGGTG
**Cavin4**	TAAAATCCGTCCAGATTGACCTG	GAGCACTAACTTTTCGGGTTTTC
**Cdc42**	CCATCGGAATATGTACCGACTG	CTCAGCGGTCGTAATCTGTCA
**Cdc42bpa**	GGTGATTGGTCGAGGAGCTTT	TCACGAAAACATGCTGTCTCAG
**Cdc42se2**	TTCTGGTTGTGTTTCAACTGCT	GGCTCTCCAATCATACTTCTGTC
**IQGAP2**	TGGTTAAGAGCGATGGAGTCT	CAGATACAAACTCAGTGCGTGA
**Rac1**	ATGTCCGTGCAAAGTGGTATC	CTCGGATCGCTTCGTCAAACA
**Rac2**	CAACGCCTTTCCCGGAGAG	TCCGTCTGTGGATAGGAGAGC
**RhoA**	GGAAAGCAGGTAGAGTTGGCT	GGCTGTCGATGGAAAAACACAT
**RhoB**	CTGCTGATCGTGTTCAGTAAGG	TCAATGTCGGCCACATAGTTC
**RhoC**	GGAGGTCTACGTCCCTACTGT	CGCAGTCGATCATAGTCTTCC
**RhoE/Rnd3**	TTACACGGCCAGTTTTGAAATCG	GGGCGGACATTGTCATAGTAAG
**RhoJ**	AGGGGCAACGACGAGAAGA	TTGGCGTAGCTCATCAGCAG

Supplementary Table 2. List of primers used for qRT-PCRs

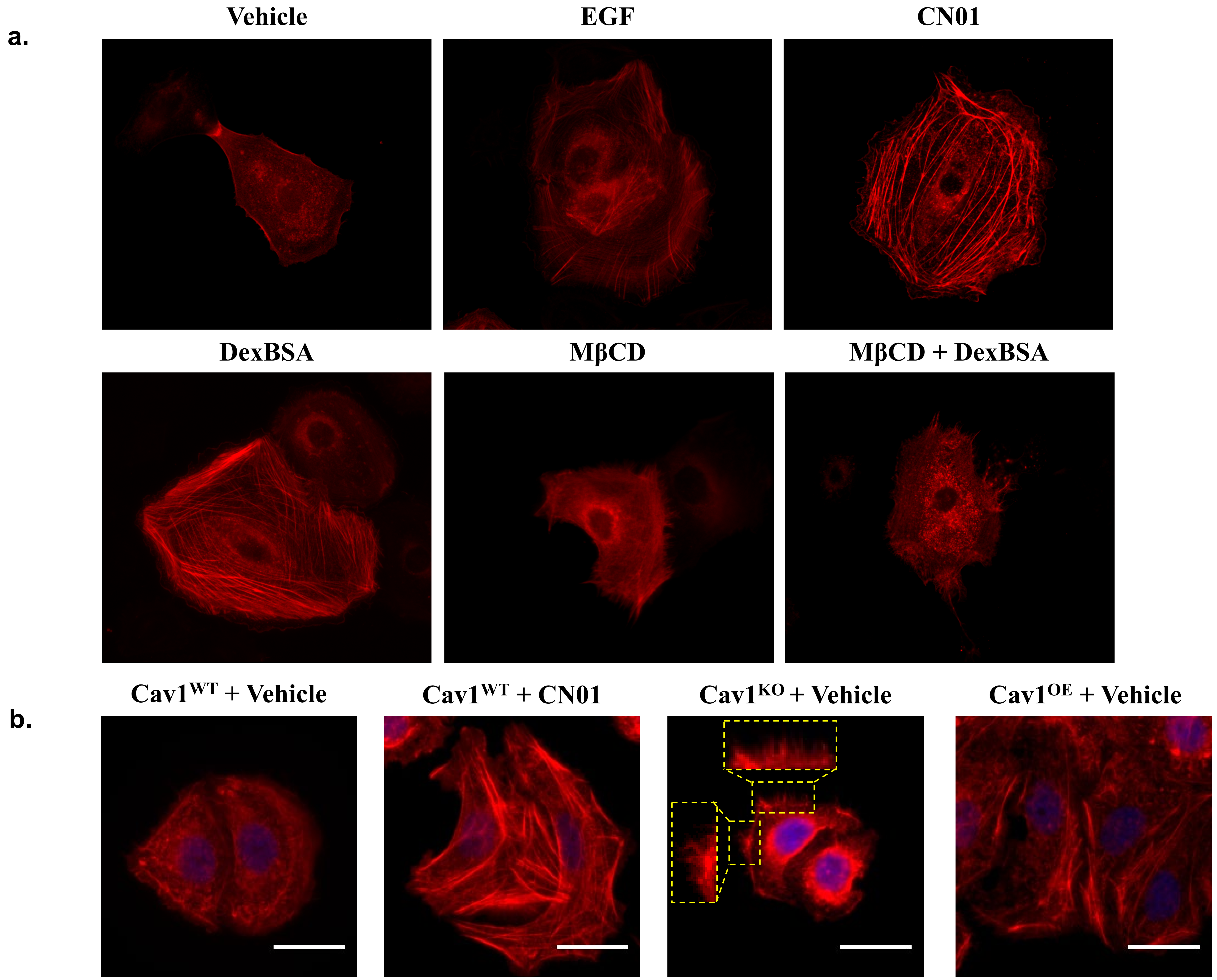

Supplementary Figure 1. Glucocorticoids alter cytoskeletal remodeling and promote increased formation stress fibers in a Cav1-dependend manner. a) Primary human keratinocytes treated with Vehicle, DexBSA, CN01 (Calpeptin) in presence/absence of MβCD and then subject to phalloidin staining. b) Normal HaCaT keratinocytes expressing endogenous levels of Cav1 ($Cav1^{WT}$), Cav1 knockout ($Cav1^{KO}$) and Cav1 overexpressing ($Cav1^{OE}$) cells were treated with -/+ CN01. Insets show presence of filopodial protrusions in $Cav1^{KO}$ keratinocytes. Scale bar = 25um.

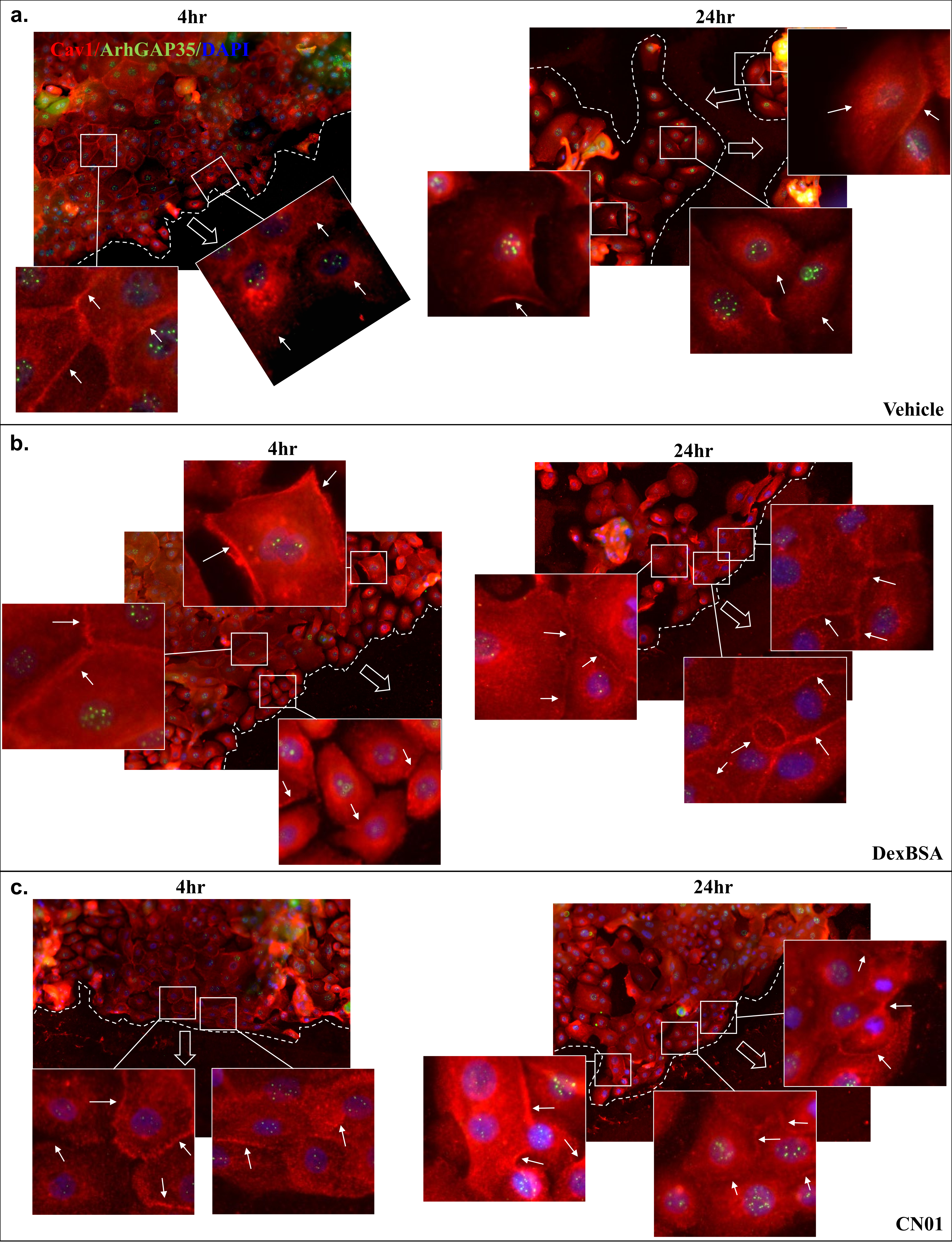

Supplementary Figure 2. Glucocorticoids induce mislocalization of Cav1 during cell migration.

Supplementary Figure 2. Glucocorticoids induce mislocalization of Cav1 during cell migration. Primary human keratinocytes were subject to wound scratch assay, fixed at 4hrs and 24hrs after wound induction and immunostained with Cav1 and ArhGAP35 in presence/absence of DexBSA. a) Vehicle treated cells exhibit localization of Cav1 at the cell membrane away from the migrating edge, whereas migrating cells exhibit polarized localization of Cav1 and a diminished localization at the cell membrane. b) DexBSA treated cells retard cell migration in part by mislocalizing Cav1 to the cell membrane, similarly to CN01 treated cells (c). Dashed lines correspond to the wound edge; open arrows correspond to the direction of migration; filled arrows point to localization of Cav1 within a particular set of cells. DexBSA-BSA conjugated dexamethasone, CN01-calpeptin, an activator of RhoA.

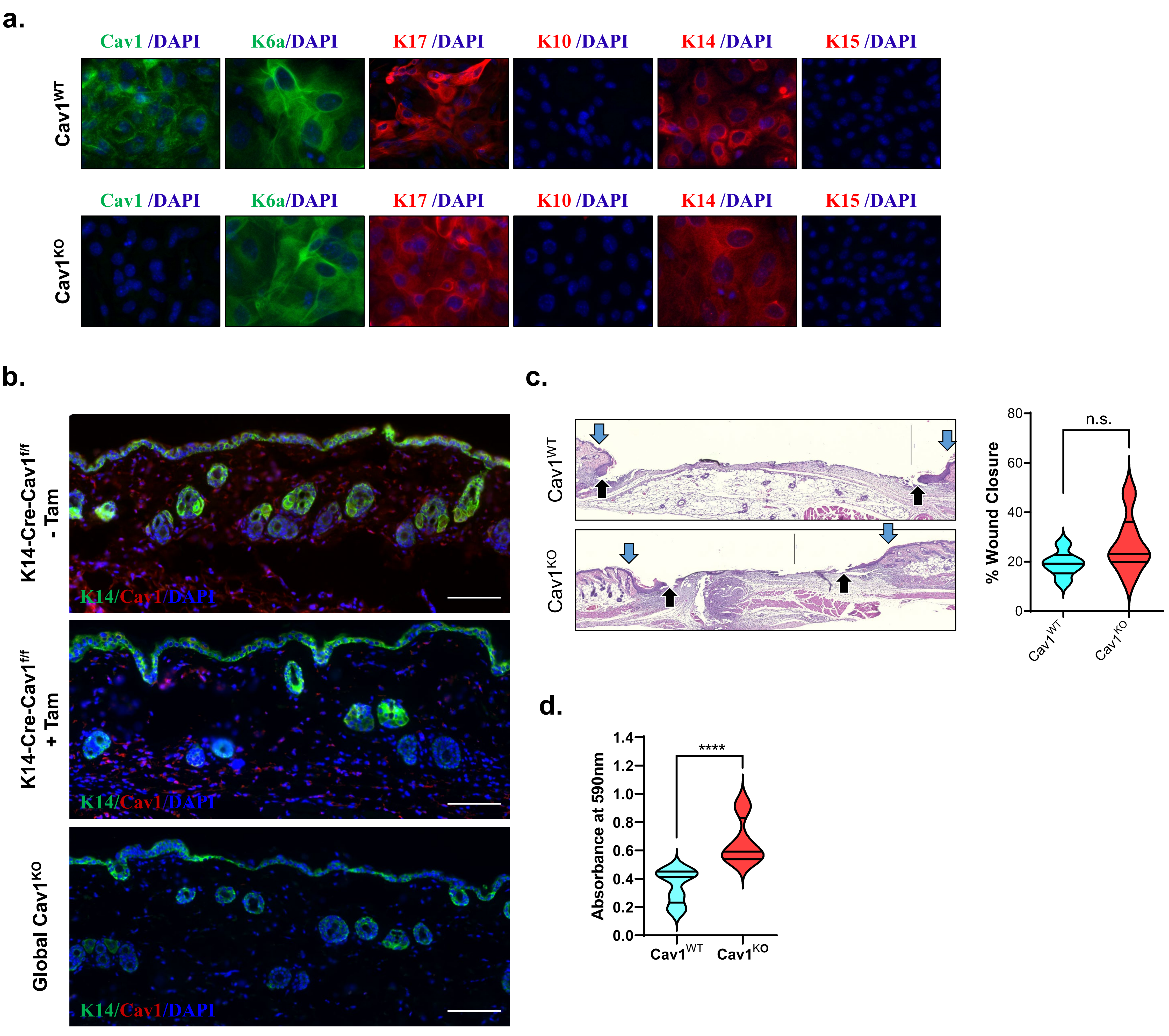

Supplementary Figure 3. Immunofluorescence staining of murine skin explants. a) Mouse explants from C57BL6 (Cav1^{WT}) and global Cav1^{KO} mice were confirmed to be activated (K6/K17 positive) non-differentiating (K10 negative) basal (K14 positive, K15 negative) keratinocytes. b) Confirmation of Cav1 knockdown in mouse models used during wound healing studies. K14cre-Cav1$^{f/f}$ mice treated with tamoxifen (tam) exhibit selective ablation of Cav1 from epidermal keratinocytes and presence of Cav1 in the dermis. c) Global Cav1 knockout mice exhibit no difference in wound closure in comparison to their Cav1wt counter mates. d) Quantification of cell proliferation at the completion of explant migration assay. Cells were stained with crystal violet, eluted with 10% (v/v) acetic acid and quantified by measuring absorbance at 590nm, confirming previously reported observation that Cav1^{KO} cells exhibit increased proliferation.

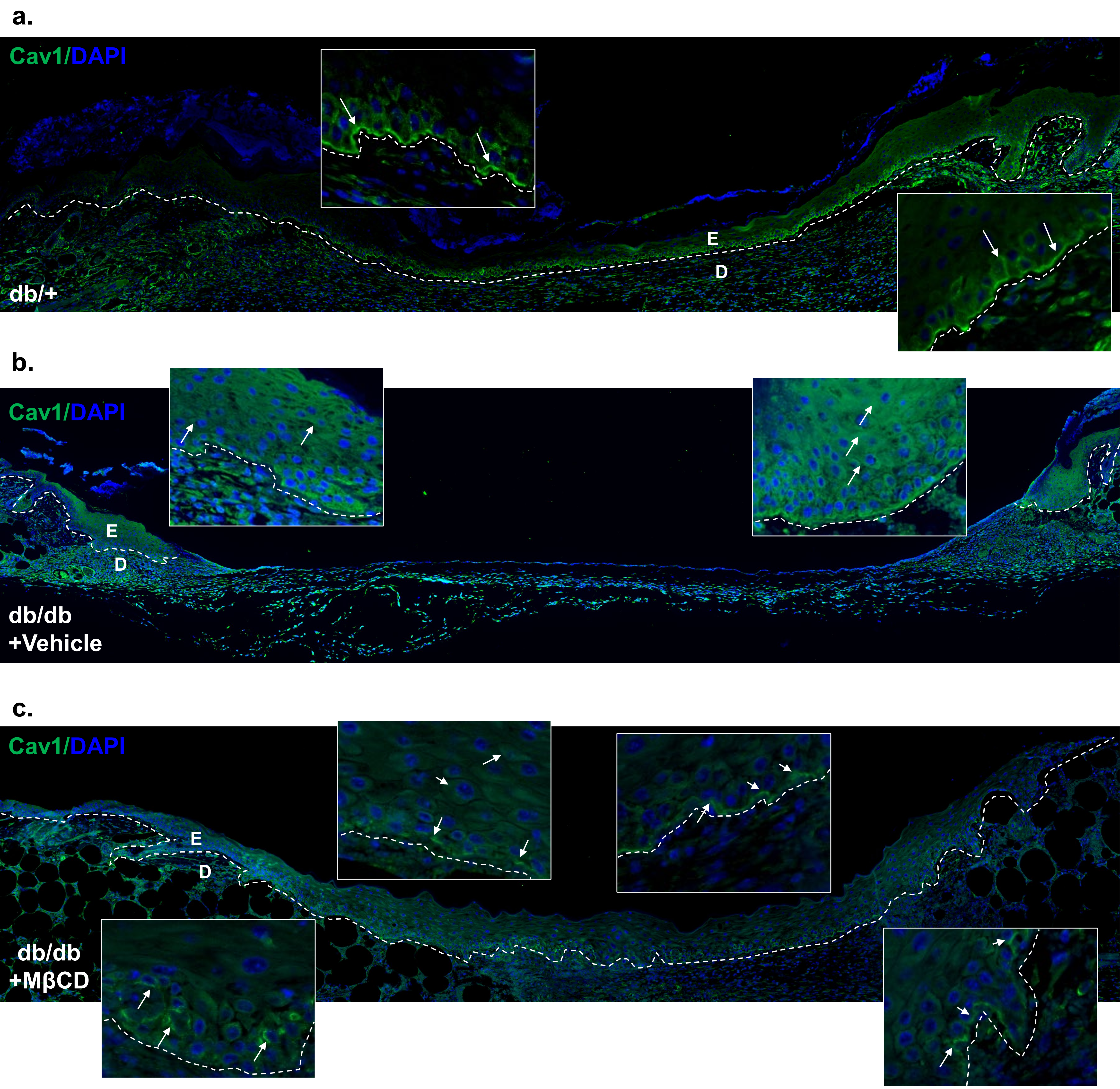

Supplementary Figure 4. Cav1 localization during acute wound healing. Day 5 wounds from db/+, db/db and db/db + MβCD were subject to Cav1 immunostaining. a) Db/+ mice exhibited full wound closure with Cav1 localizing primarily to the basal keratinocytes as previously reported. b) Vehicle treated db/db mice exhibited only partial wound closure with Cav1 mislocalizing to both basal and suprabasal layers of the epidermis. c) MβCD treated db/db mice reversed the inhibition of wound closure seen in db/db mice with Cav1 primarily localizing to basal keratinocytes. Interestingly any suprabasal Cav1 staining was localizing to cytoplasm. E- epidermis; D- dermis.

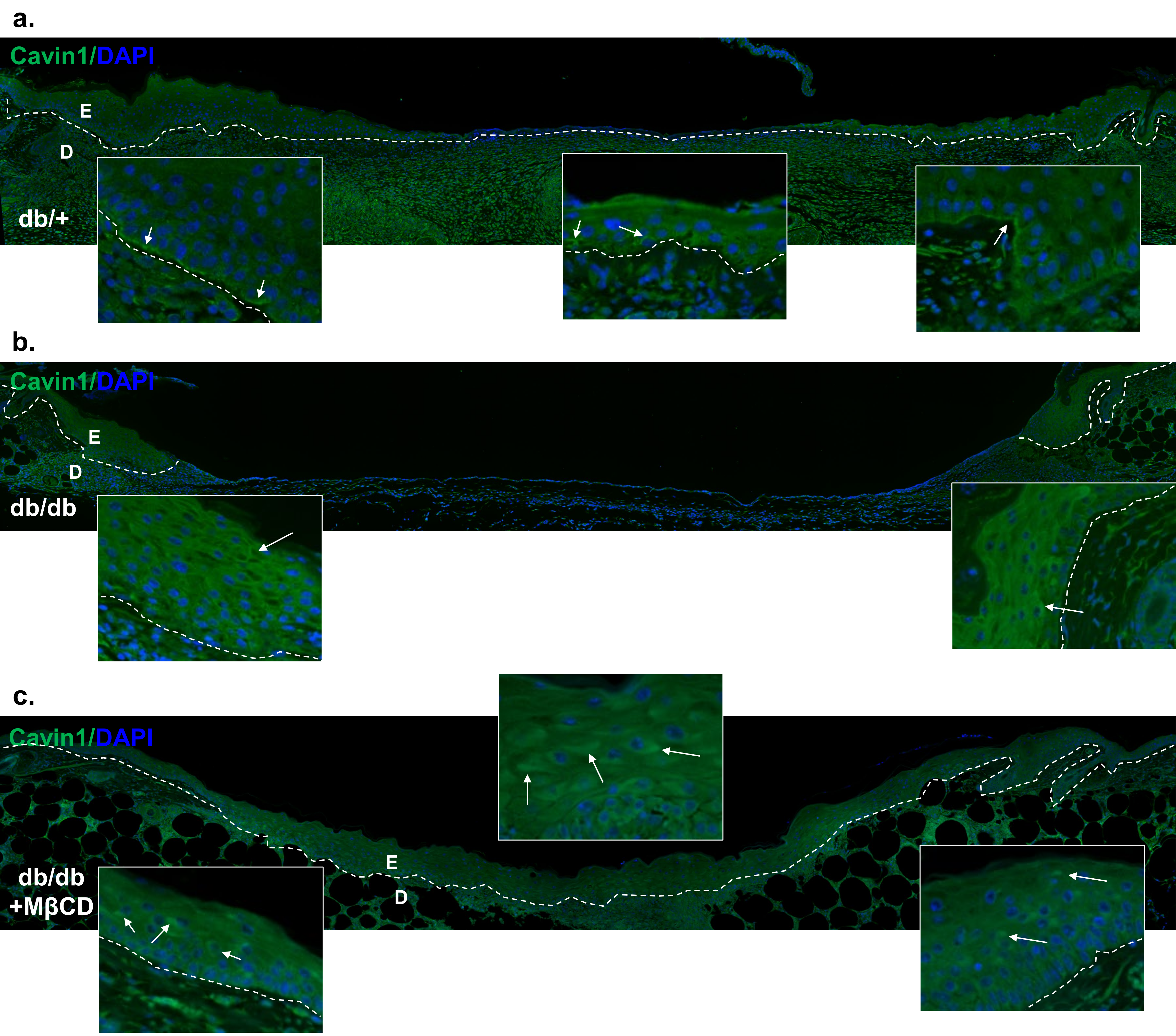

Supplementary Figure 5. Cavin1 localization during acute wound healing. Day 5 wounds from db/+, db/db and db/db + MβCD were subject to Cavin1 immunostaining. a) Db/+ mice exhibited full wound closure with Cavin1 localizing to both basal and suprabasal layers of the epidermis. b) Vehicle treated db/db mice exhibited only partial wound closure with Cavin1 localizing to both basal and suprabasal layers of the epidermis, however majority of the staining was membranous. c) MβCD treated db/db mice reversed the inhibition of wound closure seen in db/db mice with Cavin1 localizing to both basal and suprabasal epidermis. Interestingly, there was a lot of Cavin1 localizing to the cytoplasm corresponding to perturbation of Caveolae by MβCD. E- epidermis; D- dermis.

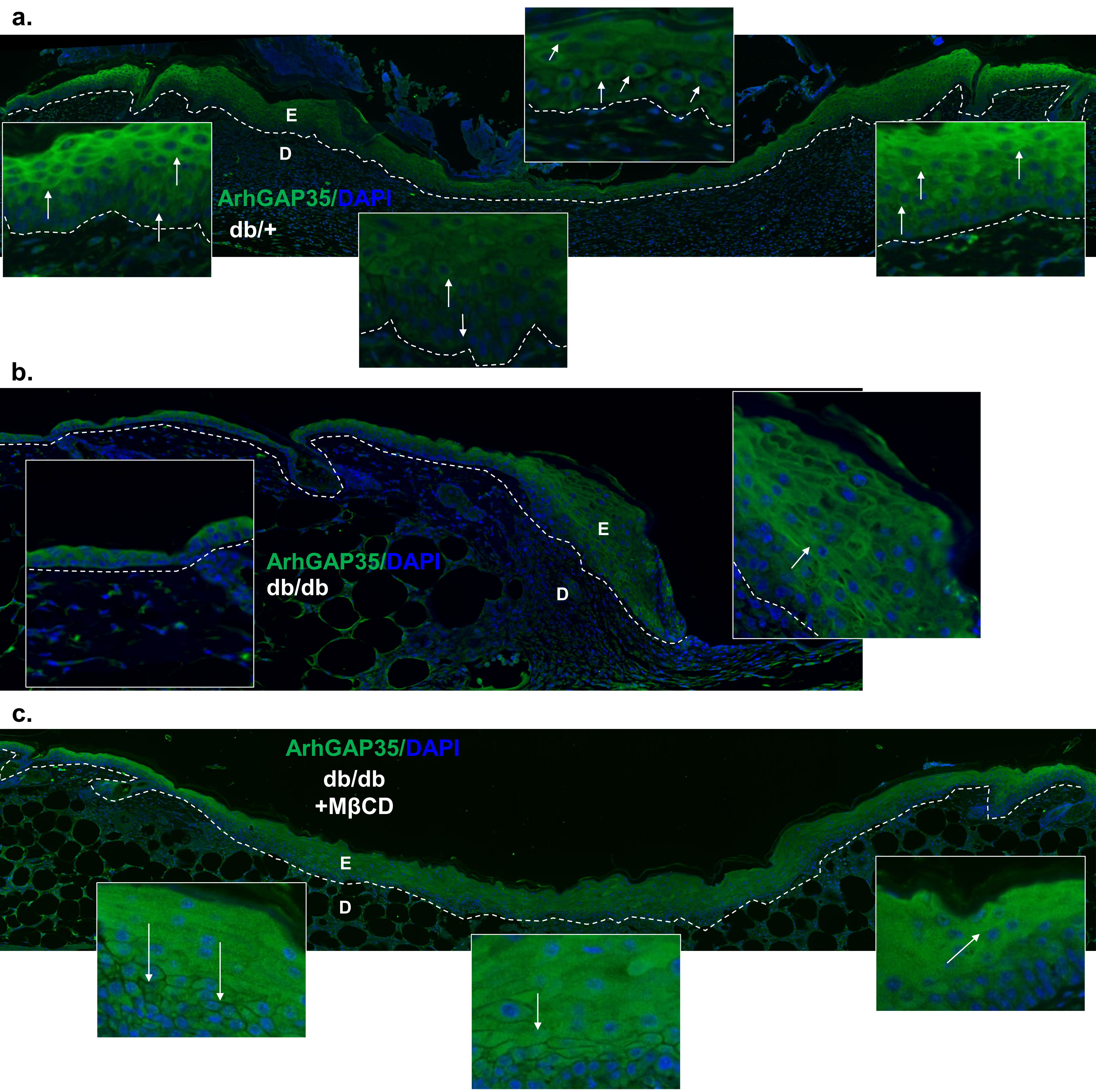

Supplementary Figure 6. ArhGAP35 localization during acute wound healing. Day 5 wounds from db/+, db/db and db/db + MβCD were subject to ArhGAP35 immunostaining. a) Db/+ mice exhibited full wound closure with ArhGAP35 localizing to primarily to suprabasal layers of the epidermis, which are devoid of Cav1 staining. b) Vehicle treated db/db mice exhibited only partial wound closure with ArhGAP35 localizing to both basal and suprabasal layers of the epidermis, however majority of the staining was membranous. c) MβCD treated db/db mice reversed the inhibition of wound closure seen in db/db mice with ArhGAP35 localizing to both basal and suprabasal epidermis. Interestingly, majority of ArhGAP35 localized to the cytoplasm. E- epidermis; D- dermis.

Supplementary Figure 7. Uncropped images of full western blots corresponding to each of the figures in the main text.

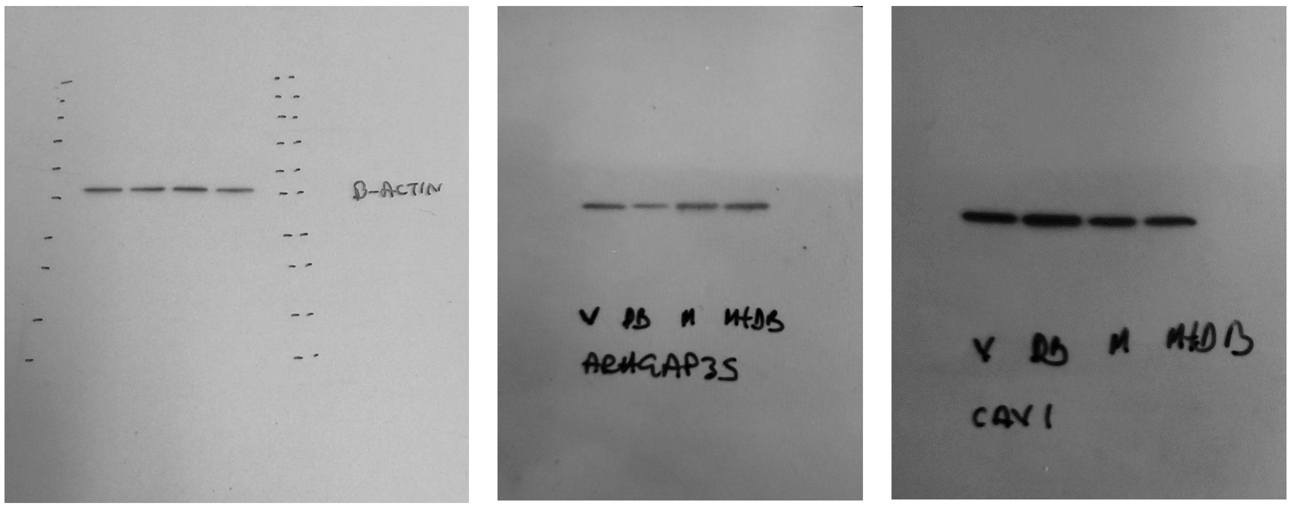

Fig. 2b

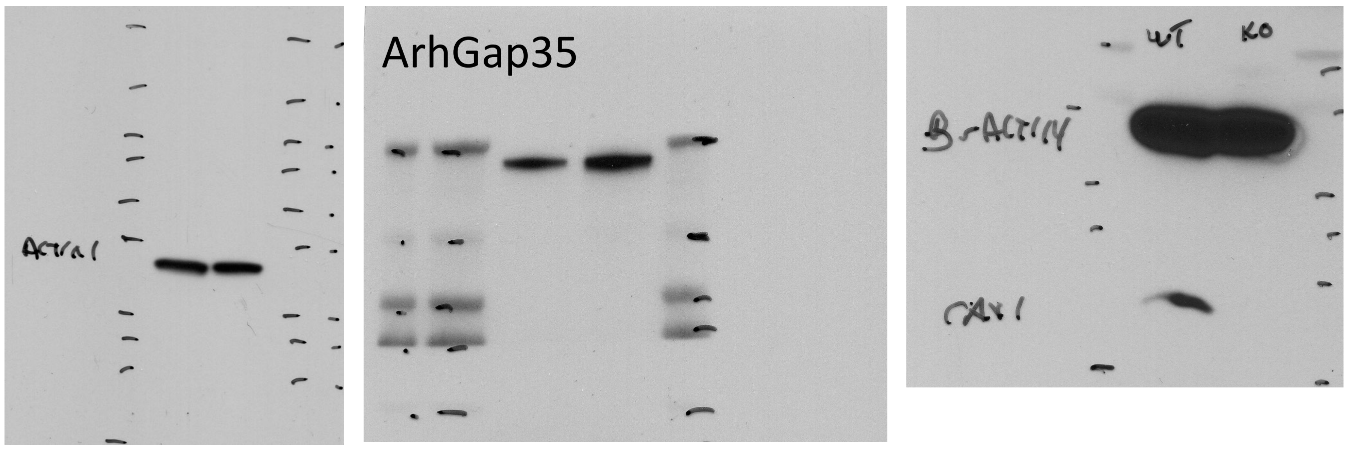

Fig. 2d- top panel

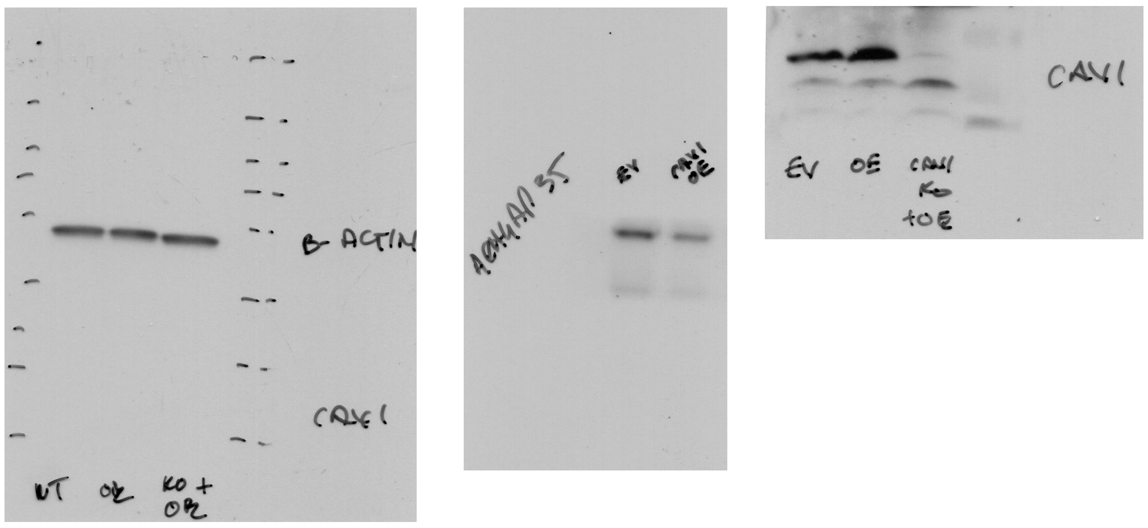

Fig. 2d- bottom panel

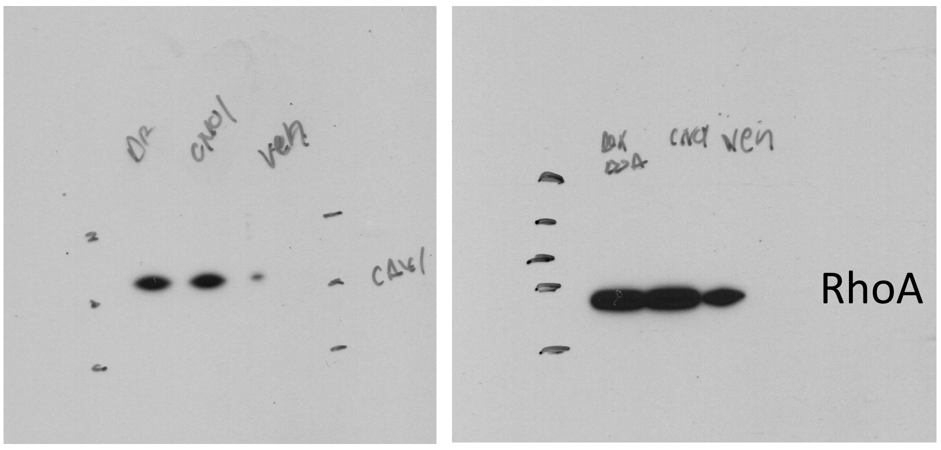

Fig. 3b – RhoA-GTP IP

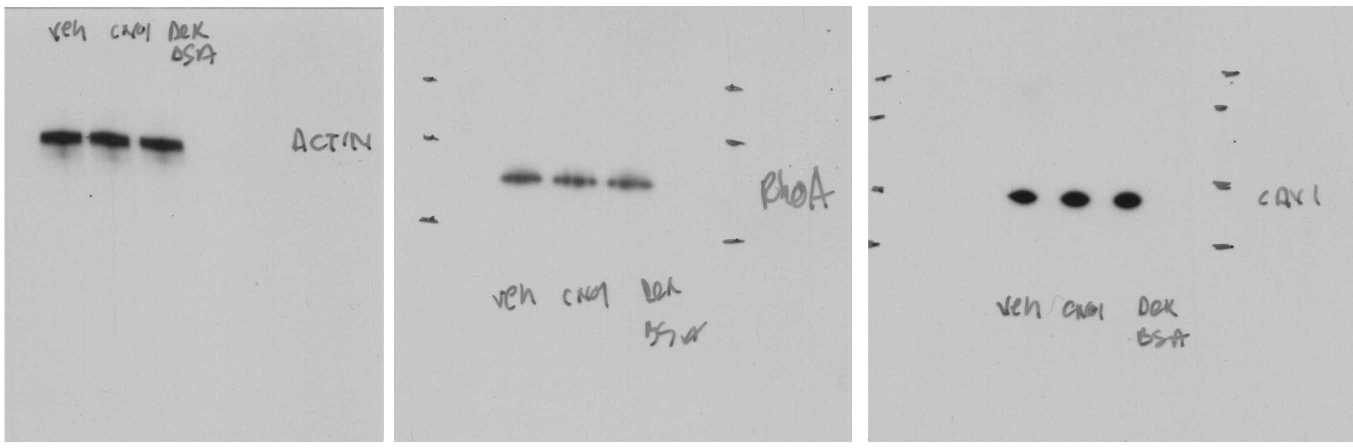

Fig. 3b – Whole Cell lysate

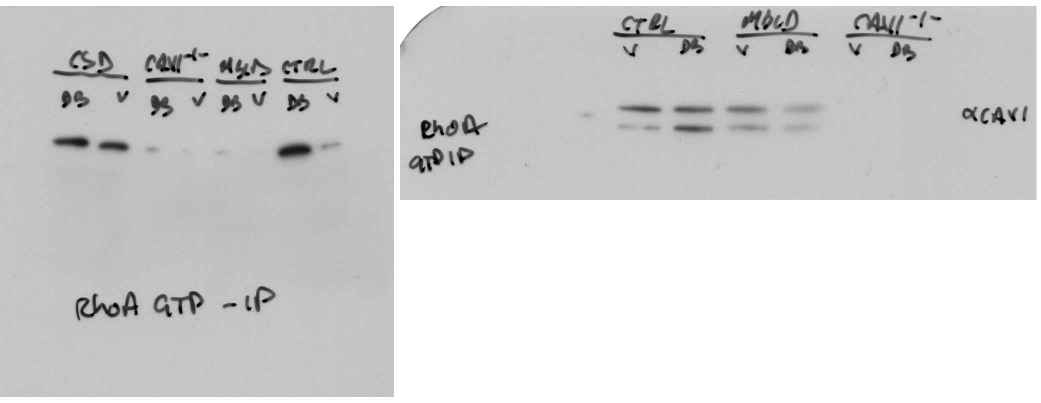

Fig. 3d – RhoA-GTP IP

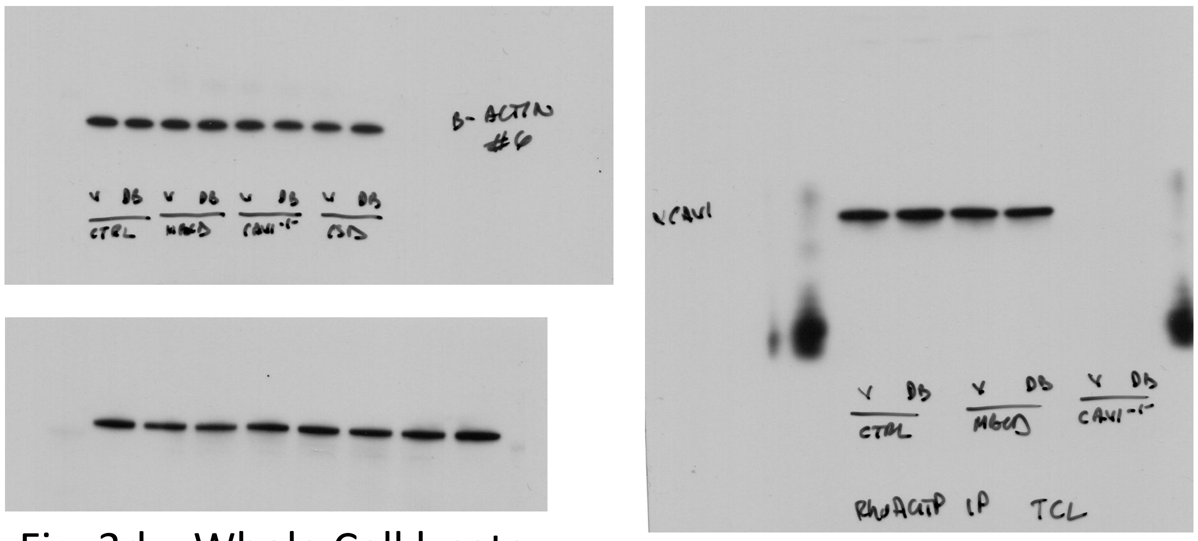

Fig. 3d – Whole Cell lysate

Supplementary Figure 7. Uncropped images of full western blots corresponding to each of the figures in the main text.

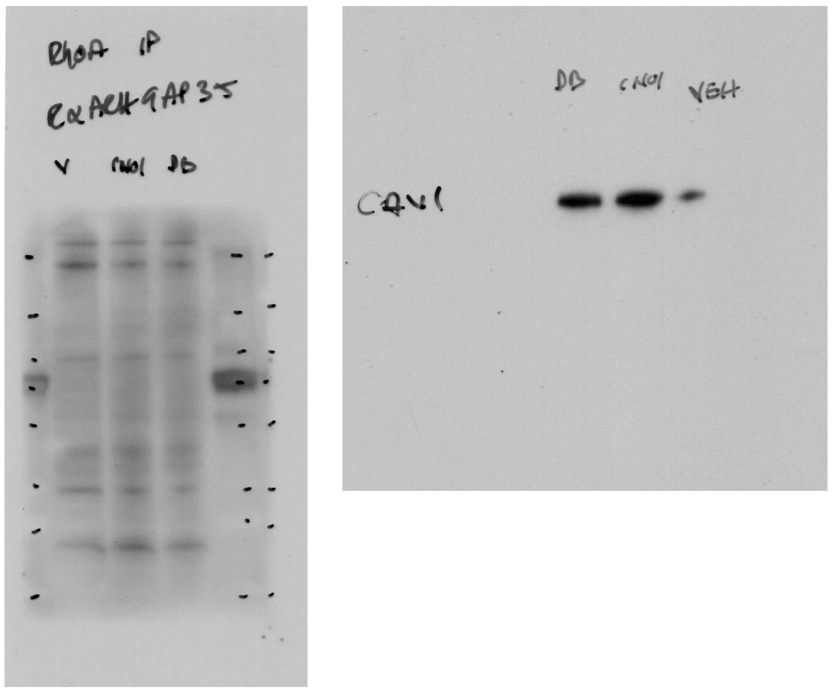

Fig. 3e – RhoA-IP

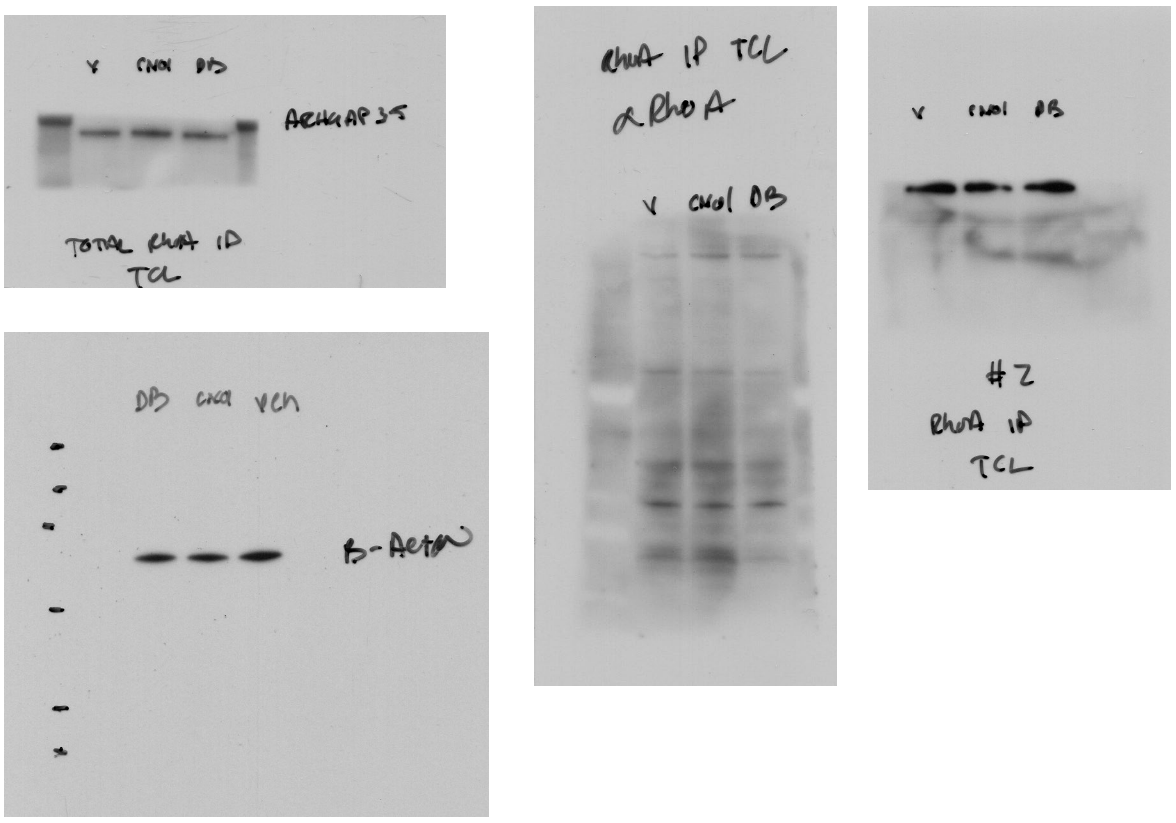

Fig. 3e – Whole Cell lysate

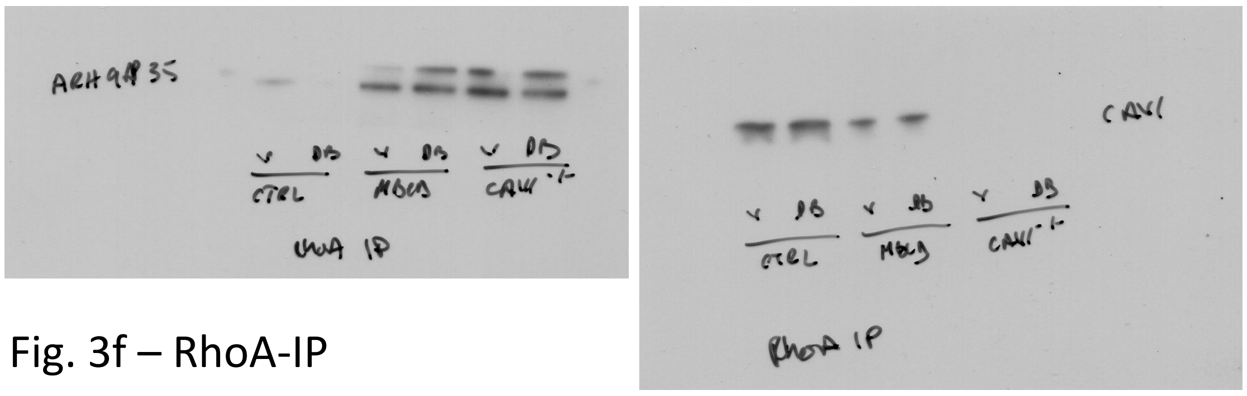

Fig. 3f – RhoA-IP

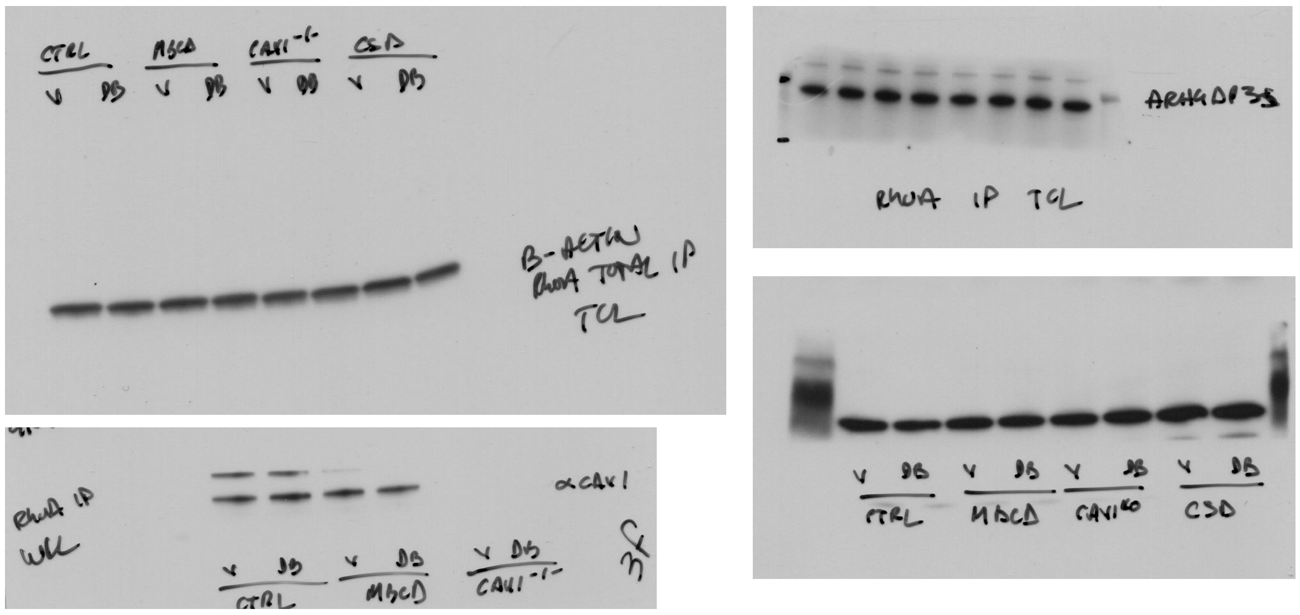

Fig. 3f – Whole Cell lysate

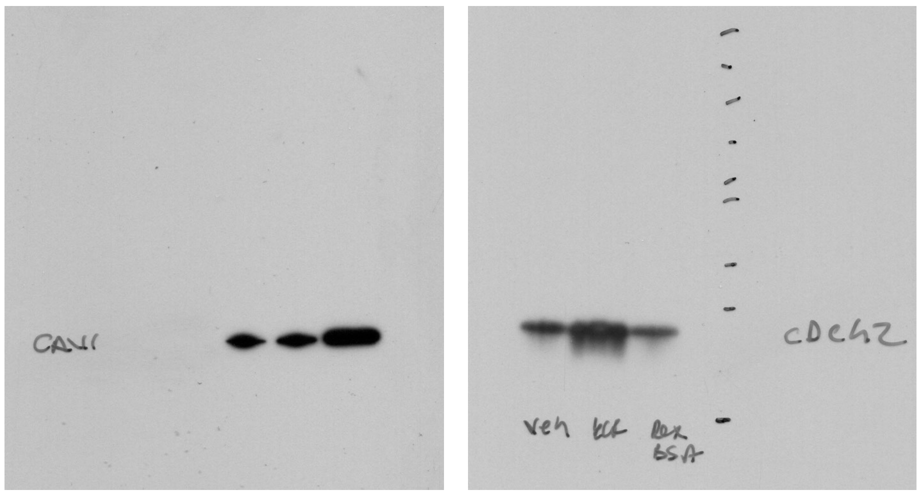

Fig. 4b – Cdc42-GTP-IP

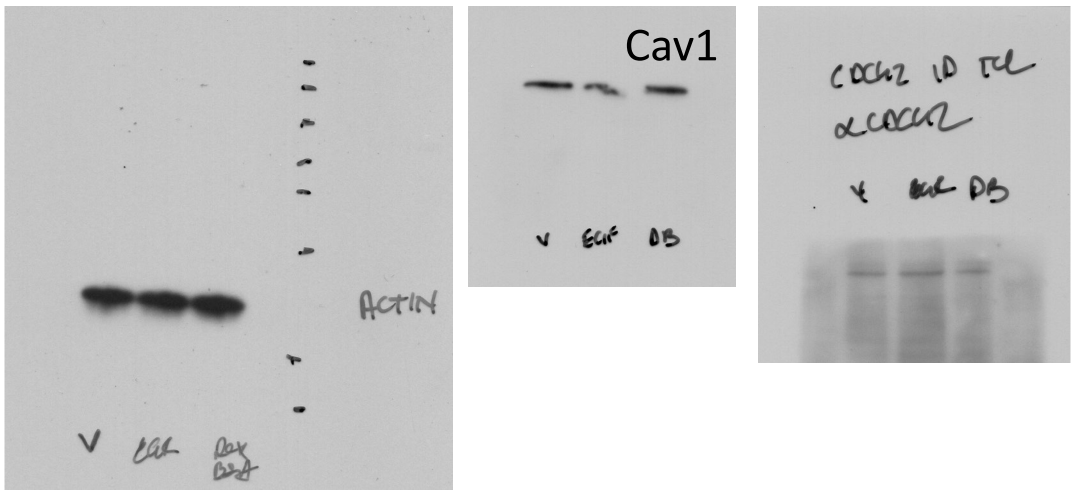

Fig. 4b – Whole Cell lysate

Supplementary Figure 7. Uncropped images of full western blots corresponding to each of the figures in the main text.

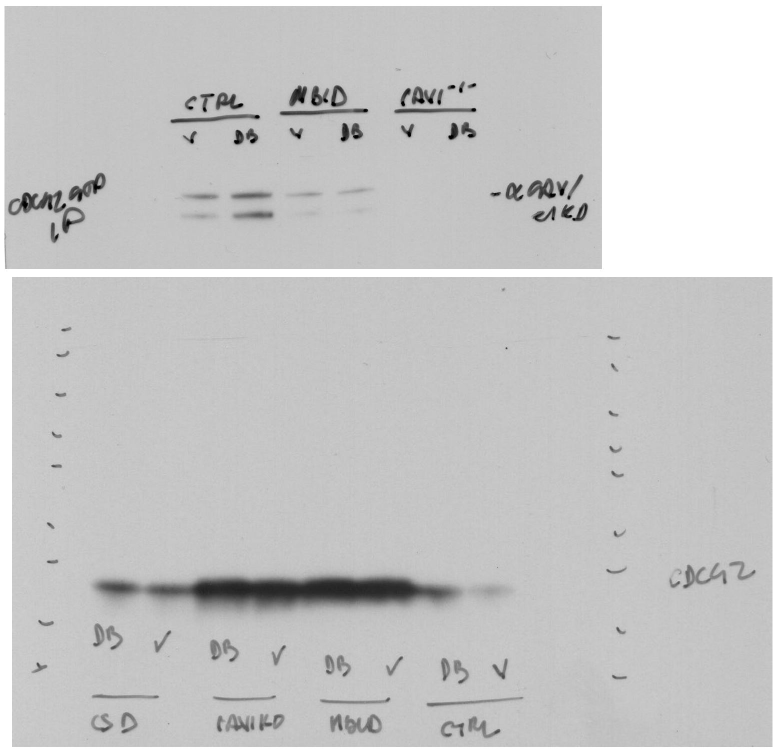

Fig. 4d – Cdc42-GTP-IP

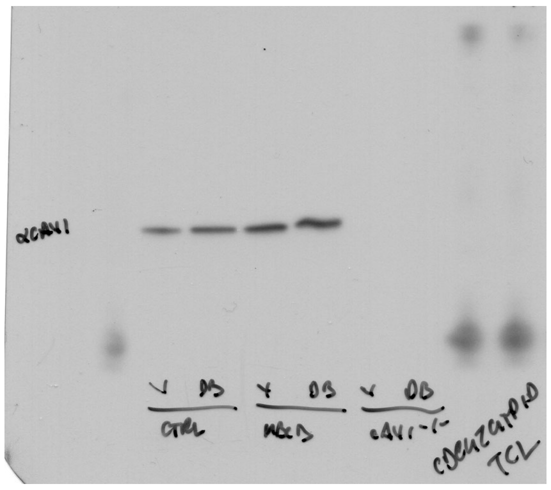

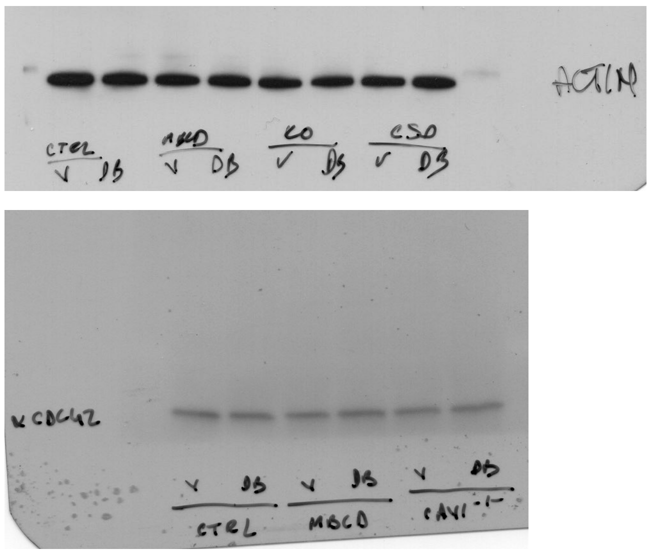

Fig. 4d – Whole Cell lysate

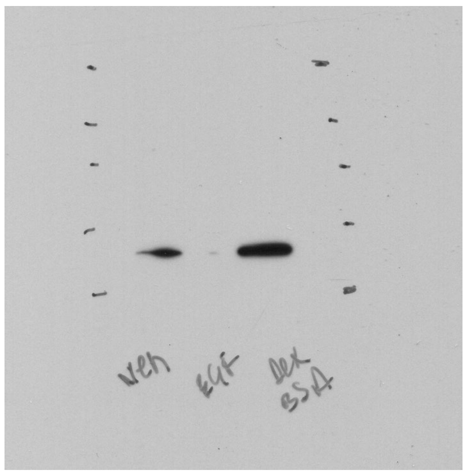

Fig. 4e – Cdc42-GTP-IP

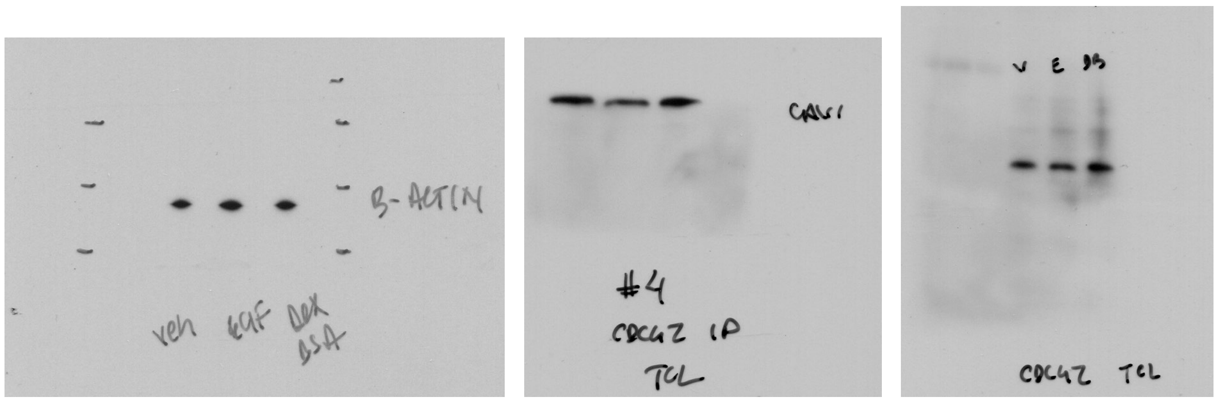

Fig. 4e – Whole Cell lysate

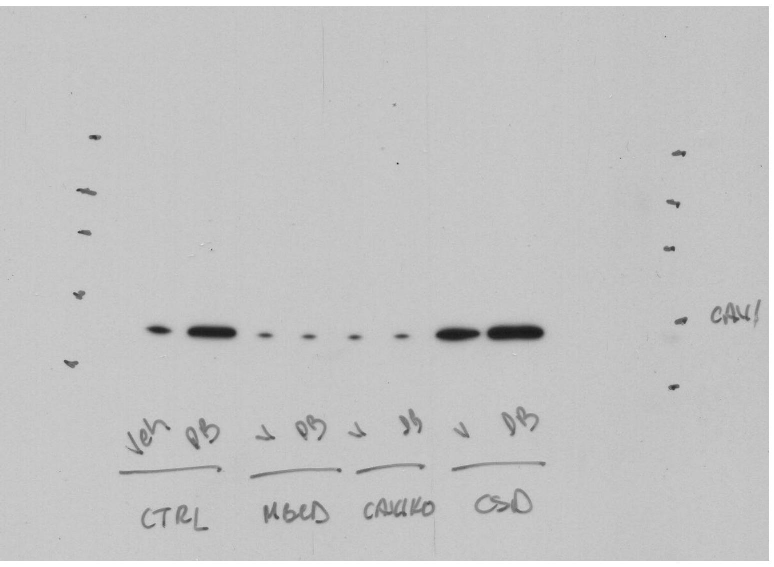

Fig. 4f – Cdc42-GTP-IP

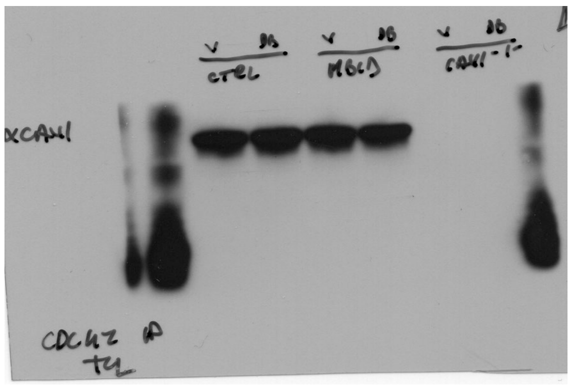

Fig. 4f – Whole Cell lysate

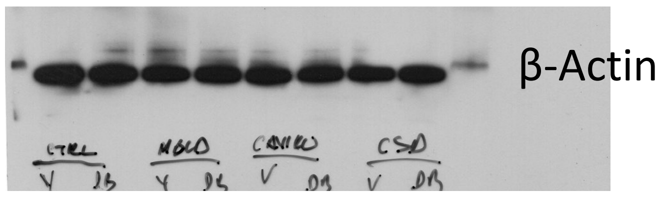

β-Actin

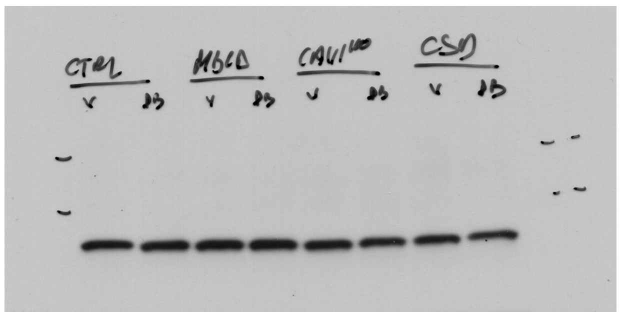

Cdc42
