# Supplementary material for: Glucocorticoid-mediated induction of caveolin-1 disrupts cytoskeletal organization, inhibits cell migration and re-epithelialization of non-healing wounds
Source: Commun Biol. 2021 Jun 18;4:757. doi: 10.1038/s42003-021-02298-5 (PMC8213848; doi:10.1038/s42003-021-02298-5)
Supplement: Supplementary file 4 — Reporting Summary [file 42003_2021_2298_MOESM4_ESM.pdf]

nature research

Corresponding author(s): Jozic, Ivan; Tomic-Canic, Marjana

Last updated by author(s): Mar 18, 2021

Reporting Summary

Nature Research wishes to improve the reproducibility of the work that we publish. This form provides structure for consistency and transparency in reporting. For further information on Nature Research policies, see our Editorial Policies and the Editorial Policy Checklist.

Statistics

For all statistical analyses, confirm that the following items are present in the figure legend, table legend, main text, or Methods section.

n/a	Confirmed	
☐	☒	The exact sample size (*n*) for each experimental group/condition, given as a discrete number and unit of measurement
☐	☒	A statement on whether measurements were taken from distinct samples or whether the same sample was measured repeatedly
☐	☒	The statistical test(s) used AND whether they are one- or two-sided<br>*Only common tests should be described solely by name; describe more complex techniques in the Methods section.*
☒	☐	A description of all covariates tested
☒	☐	A description of any assumptions or corrections, such as tests of normality and adjustment for multiple comparisons
☐	☒	A full description of the statistical parameters including central tendency (e.g. means) or other basic estimates (e.g. regression coefficient) AND variation (e.g. standard deviation) or associated estimates of uncertainty (e.g. confidence intervals)
☒	☐	For null hypothesis testing, the test statistic (e.g. F, t, r) with confidence intervals, effect sizes, degrees of freedom and P value noted<br>*Give P values as exact values whenever suitable.*
☒	☐	For Bayesian analysis, information on the choice of priors and Markov chain Monte Carlo settings
☒	☐	For hierarchical and complex designs, identification of the appropriate level for tests and full reporting of outcomes
☒	☐	Estimates of effect sizes (e.g. Cohen's *d*, Pearson's *r*), indicating how they were calculated

Our web collection on statistics for biologists contains articles on many of the points above.

Software and code

Policy information about availability of computer code

Data collection	*Provide a description of all commercial, open source and custom code used to collect the data in this study, specifying the version used OR state that no software was used.*
Data analysis	*Provide a description of all commercial, open source and custom code used to analyse the data in this study, specifying the version used OR state that no software was used.*

For manuscripts utilizing custom algorithms or software that are central to the research but not yet described in published literature, software must be made available to editors and reviewers. We strongly encourage code deposition in a community repository (e.g. GitHub). See the Nature Research guidelines for submitting code & software for further information.

Data

Policy information about availability of data

All manuscripts must include a data availability statement. This statement should provide the following information, where applicable:

- Accession codes, unique identifiers, or web links for publicly available datasets
- A list of figures that have associated raw data
- A description of any restrictions on data availability

All data associated with this study are available in the main text or the supplementary materials.

April 2020

Field-specific reporting

Please select the one below that is the best fit for your research. If you are not sure, read the appropriate sections before making your selection.

- [x] Life sciences
- [ ] Behavioural & social sciences
- [ ] Ecological, evolutionary & environmental sciences

For a reference copy of the document with all sections, see nature.com/documents/nr-reporting-summary-flat.pdf

Life sciences study design

All studies must disclose on these points even when the disclosure is negative.

Sample size	Sample size was determined based on previously published works from both our lab and others.
Data exclusions	No data has been excluded
Replication	All experiments were performed repeated at least 3 times ensuring that data is reproducible. Number of both technical and biological replicates is indicated in each figure.
Randomization	Randomization does not apply to any experiments where biomaterials are derived from the same biological replicate, i.e., one piece of skin coming from a single skin donor undergoing routine reduction surgeries divided into multiple treatments, or organotypic skin equivalents that are made from same batch of cells (both keratinocytes and fibroblasts) isolated from the same individual. To ensure reproducibility and robustness of collected data, sequential experiments were completed using different donors. Hence, randomization is not relevant to majority of the data utilized in this study. Where survival surgeries were completed on mouse models, once correct genotype was confirmed, age/sex-matched litter mates were randomly assigned to either control or treatment group, using a random number generator.
Blinding	Investigators were blinded during analysis of wound re-epithelialization, where samples were coded and wound re-epithelialization measurements completed by three independent investigators.

Reporting for specific materials, systems and methods

We require information from authors about some types of materials, experimental systems and methods used in many studies. Here, indicate whether each material, system or method listed is relevant to your study. If you are not sure if a list item applies to your research, read the appropriate section before selecting a response.

Materials & experimental systems

n/a	Involved in the study	
☐	☒	Antibodies
☐	☒	Eukaryotic cell lines
☒	☐	Palaeontology and archaeology
☐	☒	Animals and other organisms
☐	☒	Human research participants
☒	☐	Clinical data
☒	☐	Dual use research of concern

Methods

n/a	Involved in the study	
☒	☐	ChIP-seq
☒	☐	Flow cytometry
☒	☐	MRI-based neuroimaging

Antibodies

Antibodies used	All of the antibodies used in the study are listed in the Antibodies and reagents tab of the Materials and Methods section including concentration used, supplier name and catalog number.
Validation	Antibodies were selected based on 1) published reports of specificity using positive and negative control cells and/or tissue (e.g. knockout mice); and 2) validation data from the commercial vendor. All the antibodies that we use (for western blotting, IHC and IF) are purchased from manufacturers with hybridoma clone identification, lot number and appropriate references. We also validate results using complementary methods such as PCR. We consistently use protocols for all our experiments. As new lots of antibodies are received, they are validated by several criteria, including detection of antigen of the expected MW by western blotting.

Eukaryotic cell lines

Policy information about cell lines

Cell line source(s)	Immortalized human keratinocyte (HaCaT) cell line was purchased from AddexBio (Cat #T0002001)
Authentication	Certificate of analysis was obtained from the supplier that validates authenticity and can be shared upon request.

Mycoplasma contamination	All of the cells used were tested and confirmed to be free of mycoplasma
Commonly misidentified lines (See ICLAC register)	N/A

Animals and other organisms

Policy information about studies involving animals; ARRIVE guidelines recommended for reporting animal research

Laboratory animals	We used multiple mouse models in this study all of which were 8 week old females at the time of wounding. 1) C57BL/6J purchased from Jackson Labs (cat# 000664); 2) Cav1 global knockouts B6.Cg-Cav1tm1Mls/J purchased from Jackson Labs (cat# 007083); leptin receptor mutant db/db mice B6.BKS(D)-Leprdb/J purchased from Jackson Labs (cat# 000697); tamoxifen inducible K14cre mice Tg (KRT14-cre/ERT)20Efu/J purchased from Jackson Labs (cat# 005107); Cav1flox/flox mice C57Bl/6J-Cav1Flox were a generous gift from Timothy Thompson and Michael H. Elliot, one of the co-authors on this manuscript;
Wild animals	*Provide details on animals observed in or captured in the field; report species, sex and age where possible. Describe how animals were caught and transported and what happened to captive animals after the study (if killed, explain why and describe method; if released, say where and when) OR state that the study did not involve wild animals.*
Field-collected samples	*For laboratory work with field-collected samples, describe all relevant parameters such as housing, maintenance, temperature, photoperiod and end-of-experiment protocol OR state that the study did not involve samples collected from the field.*
Ethics oversight	All of the protocols used in this manuscript were approved by the University of Miami Institutional Animal Care and Use Committee under protocol 19-197-LF

Note that full information on the approval of the study protocol must also be provided in the manuscript.

Human research participants

Policy information about studies involving human research participants

Population characteristics	Population characteristics were included in the manuscript as supplemental figure 7
Recruitment	Inclusion criteria for DFU were (1) diabetes mellitus; (2) an ulcer on the plantar aspect of their foot that is larger than 0.5 cm2; (3) neuropathy; (4) age 21 years or older; (5) wound duration >4 weeks; (6) hemoglobin A1c: ≤13.0%. Ulcers with clinical signs of infection were excluded. Exclusion criteria for DFU were (1) active cellulitis; (2) osteomyelitis; (3) gangrene; (4) vascular insufficiency (defined as an ankle-brachial index (ABI) <0.7 and for those with an ABI >1.3; (5) revascularization to the ipsilateral lower extremity in the last 6 weeks; (6) any experimental drugs taken or applied topically to the wound for 4 weeks preceding the study. Due to limited size of human tissue samples, majority of material was utilized for the genomic and tissue analyses and are no longer available.
Ethics oversight	Control healthy human skin specimens were obtained as discarded tissue from reduction surgery procedures in accordance to institutional approvals 2 as previously described. Specifically, protocol to obtain unidentified, discarded human skin specimens from reduction surgery was submitted to University of Miami Human Subject Research Office (HSRO). Upon review conducted by University of Miami Institutional Review Board (IRB) it was determined that such protocol does not constitute Human Subject Research. As such, this project was not subject to IRB review under 45 7 CFR46.101.2. Chronic wound samples were obtained from consenting patients receiving standard care at the University Of Miami Hospital. These protocols were approved by the Institutional Review Board (IRB protocols #20150912; #20150222; #20090709, #27085).

Note that full information on the approval of the study protocol must also be provided in the manuscript.
